# A Machine Learning Model to Predict Drug Transfer Across the Human Placenta Barrier

**DOI:** 10.3389/fchem.2021.714678

**Published:** 2021-07-20

**Authors:** Juan I. Di Filippo, Mariela Bollini, Claudio N. Cavasotto

**Affiliations:** ^1^ Computational Drug Design and Biomedical Informatics Laboratory, Instituto de Investigaciones en Medicina Traslacional (IIMT), CONICET-Universidad Austral, Pilar, Argentina; ^2^ Facultad de Ciencias Biomédicas and Facultad de Ingeniería, Universidad Austral, Pilar, Argentina; ^3^ Austral Institute for Applied Artificial Intelligence, Universidad Austral, Pilar, Argentina; ^4^ Centro de Investigaciones en BioNanociencias (CIBION), Consejo Nacional de Investigaciones Científicas y Técnicas (CONICET), Buenos Aires, Argentina

**Keywords:** placenta barrier permeability, machine learning, toxicology, clearence index, fetus:mother ratio

## Abstract

The development of computational models for assessing the transfer of chemicals across the placental membrane would be of the utmost importance in drug discovery campaigns, in order to develop safe therapeutic options. We have developed a low-dimensional machine learning model capable of classifying compounds according to whether they can cross or not the placental barrier. To this aim, we compiled a database of 248 compounds with experimental information about their placental transfer, characterizing each compound with a set of ∼5.4 thousand descriptors, including physicochemical properties and structural features. We evaluated different machine learning classifiers and implemented a genetic algorithm, in a five cross validation scheme, to perform feature selection. The optimization was guided towards models displaying a low number of false positives (molecules that actually cross the placental barrier, but are predicted as not crossing it). A Linear Discriminant Analysis model trained with only four structural features resulted to be robust for this task, exhibiting only one false positive case across all testing folds. This model is expected to be useful in predicting placental drug transfer during pregnancy, and thus could be used as a filter for chemical libraries in virtual screening campaigns.

## Introduction

Drug prescribing in pregnancy remains a complex and controversial issue for both pregnant women and clinicians (Leong et al., 2019). According to the Center for Disease Control and Prevention (CDC), 9 out of 10 women take at least one medication during pregnancy; and 70% of pregnant women take at least one prescribed medication (https://www.cdc.gov/pregnancy/meds/treatingfortwo/index.html). Over the past 30 years, the use of prescription drugs during the first quarter trimester of pregnancy has increased by more than 60%. This suggests that at the beginning of pregnancy, many women either present pre-chronic conditions (e.g., pre-gestational diabetes) or develop pregnancy-specific diseases (e.g., hyperemesis gravidarum, intrahepatic cholestasis of pregnancy, HELLP syndrome) which will require the administration of medications, including those which might cause fetal toxicity or teratogenesis (Eke et al., 2020). To guarantee drug safety during pregnancy, *in vitro* and *in vivo* experimental models were developed to study the transfer and metabolism of drugs across the human placental barrier. Since the placenta is the most species-specific organ, human cell lines and tissue models are considered more appropriate than *in vivo* assays performed in rodent models for evaluating the transfer of chemicals across the human placental barrier (Giaginis et al., 2012). In this regard, the *ex vivo* human placental perfusion model, which preserves placental structural integrity, and mimics the maternal and fetal blood circulation, is more suitable (Gordon et al., 2016). Unfortunately, *in vitro* and *ex vivo* methods cannot directly predict *in vivo* outcomes, making the assessment of placental transfer difficult (Hutson et al., 2011). On the other hand, *in vivo* assays are more accurate in evaluating drug toxicity. *In vivo* data can be obtained by measuring drug concentrations in the umbilical cord blood and maternal blood at delivery (Freriksen et al., 2020). The fetal-maternal concentration ratio is a widely used indicator of placental permeability that has been applied to drug monitoring (Hutson et al., 2011). However, there is an obvious ethical barrier to develop *in vivo* studies to assess the risk of transfer of chemicals across the placental membrane from the mother to the fetus. In this scenario, there is an urgent need for an integrated approach incorporating all the range of methodologies (*in vitro*, *ex vivo*, *in silico* and *in vivo* studies) to accelerate the availability of pharmacology data in pregnant women to allow the safe and effective use of medication during this physiological state.

Several Quantitative Structure–Activity Relationship (QSAR) models have been published on this topic. Based on *ex vivo* human placental perfusion results, Giaginis et al. Giaginis et al, (2009) developed a model to predict placental transfer through the calculation of the Clearance Index (CI) values for a set of 88 compounds. Using this approach, Zhang et al. Zhang et al, (2015) estimated the placental barrier permeability, also expressed as CI values, for a set of 65 compounds. Takaku et al. Takaku et al, (2015) developed a QSAR model for predicting the *in vivo* fetal–maternal blood concentration ratio (F/M ratio) for a set of 55 compounds. Later, Wang et al., using the same chemical library of 55 compounds as Takaku et al. Takaku et al, (2015), developed a QSAR model following the Organization for Economic Co-operation and Development (OECD) guidelines based on multiple linear regression adjustments for predicting *in vivo* log (F/M) values (Wang et al., 2020). These studies achieved a reasonable predictive potential (the correlation between measured and predicted values is acceptable); however, all of them were validated with few samples. Giagnis et al. used only nine compounds as a test set, Takaku et al. and Wang et al. used a test set of 14 compounds, and Zhang et al. utilized 19 compounds for the test set. Takaku et al. used three features for their QSAR model, and Wang et al. utilized two descriptors, which is a reasonable approach taking into account the number of samples in their set; however, Zhang et al. utilized 48 descriptors to construct their QSAR model.

In this study, we used available information on drug placental transfer to train machine learning (ML) algorithms in order to carry out the *in silico* prediction of whether a compound will cross the placental barrier or not. ML approaches have been consistently implemented in the last decade with different degrees of success in the drug discovery pipeline (Carpenter et al., 2018; Chen et al., 2018; Chan et al., 2019; Mak and Pichika, 2019; Cavasotto and Di Filippo, 2021a); while a ML model would not necessarily provide a clearer understanding of why some drugs cross or do not cross the placental barrier, its importance lies on the direct use for practical purposes, namely, serving as a filter in a high throughput screening campaign of a chemical library. To this purpose, we compiled a database of 248 compounds, collecting for each compound its CI value, and/or F/M ratio, and/or assessment from the literature whether it crosses or not the placental barrier. Considering the variability of the experimental parameters collected between different laboratories (Hutson et al., 2011), we decided to label each compound in a binary fashion according to whether it crosses (C) or does not cross (NC) the placental membrane, using the above mentioned information and based on a proposed set of criteria (see Methods). We used molecular descriptors as inputs and the binary output (C/NC) to train the ML classifiers to predict whether a molecule will cross the placental barrier or not. After an extensive feature selection process and the evaluation of different models, we present in this work a robust LDA classifier trained with only four features that exhibits an excellent performance. Furthermore, the model exhibits a critical characteristic, namely, the amount of molecules that cross the placenta that are misclassified as not crossing is almost null.

## Materials and Methods

### Data Collection

We collected a dataset of 248 molecules with at least one of these pieces of information: CI, F/M ratio (F/M), evidence from the literature that the molecule crosses or not the placenta barrier (Supplementary Table S1). If F/M ≤ 0.15, the molecule was labeled as NC; if F/M ≥ 0.3, the molecule was labeled as C; to avoid dubious cases, molecules in the range 0.15 < F/M < 0.3 were not included in the set. In cases where only the CI value was available, the molecule was labeled as C if CI > 0.80 (this threshold was chosen based on the fact that whenever both F/M and CI values were available, all molecules with CI > 0.8 have F/M > 0.3, i.e., they were labeled as C). If F/M ≥ 0.3 and CI < 0.8 the molecule was labeled as C, since we privileged results from *in vivo* assays over those using the perfusion method. Several molecules lacked of F/M and CI values, but evidence was found in the literature to classify them as C or NC (cf. Supplementary Table S1). Using these criteria, the dataset contained 213 molecules (∼86%) that cross the placental barrier, and 35 (∼14%) that do not. Following the standard convention, we defined the larger class as the negative one.

### Dataset Split

**FIGURE 1:**
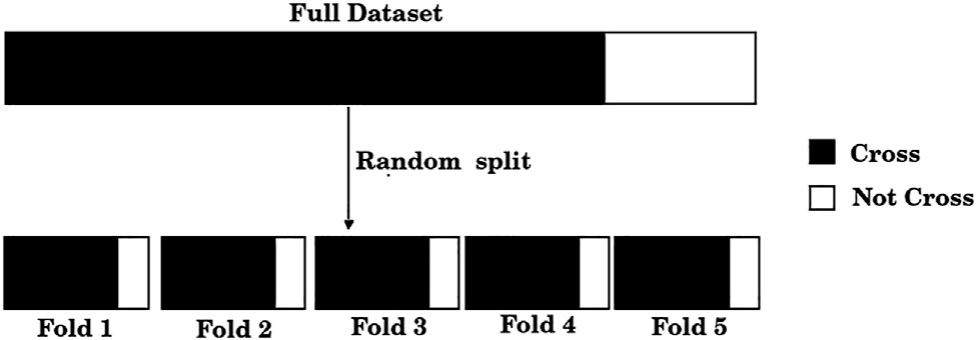
The dataset of 248 compounds was divided randomly into five folds. Each of these folds presents, approximately, the same distribution of positive and negative samples as the full dataset.

The standard training set/test set split is useful only for large size datasets, which is clearly not our case. If, for example, 20% of the dataset were used for testing, results would be reported only over 50 samples; furthermore, the results could be biased due to the unique random split of the training and test sets. Instead, we adopted a standard procedure when dealing with small datasets, a 5-fold cross-validation scheme. For this purpose, the dataset was split randomly into five folds, where each fold approximately exhibits the C/NC distribution of the entire dataset, as shown in Figure 1. Unlike a single training set/test set split, this scheme allows the use of each of the samples both in the training set (four times) and in the test set (once).

### Molecular Descriptors

Molecules were protonated at physiological pH using the ICM software (MolSoft, San Diego, CA, 2019) (Abagyan et al., 1994), in a similar fashion as in earlier works (Cavasotto and Aucar, 2020; Cavasotto and Di Filippo, 2021b), and then each molecule was visually inspected. To generate model inputs, molecules were described using a set of 5,379 features, which are summarized in Table 1. These were calculated with OpenBabel (O’Boyle et al., 2008; O’Boyle et al., 2011) and PaDEL (Yap, 2011), and included both physicochemical properties and substructure fingerprint counts. These fingerprint count features encompass electro-topological state indices (Hall and Kier, 1995), the presence of SMARTS patterns (Klekota and Roth, 2008), and the presence of chemical substructures.

**TABLE 1:** Molecular features calculated with OpenBabel and PaDEL. Physicochemical properties include classical descriptors such as molecular weight, rotatable bonds, number of Hydrogen bond donors and acceptors, etc.

Source	Name	Descriptors	Number
OpenBabel	1D and 2D descriptors	Physicochemical properties	13
PaDEL	EState fingerprints	Electrotopological state indices	79
PaDEL	KlekotahRoth fingerprints	Presence of SMARTS patterns	307
PaDEL	Substructure fingerprints	Presence of chemical substructures	4,860

### Evaluation Metrics

A binary classifier predicts all the instances as either positive 1) or negative (0). Considering that these instances can be classified correctly or incorrectly, four types of outcomes can be distinguished: True Positives (TP), True Negatives (TN), False Positives (FP), and False Negatives (FN). In general, classification algorithms predict the probability that an observation will belong to the positive class, i.e., will be 1. To make discrete predictions based on the probability provided by the classifier, that is to say, to have a binary outcome, it is necessary to define a threshold: Probabilities below this threshold are discretized as 0 and above the threshold as 1.

The Accuracy (*A*) is the percentage of accurate predictions, and is defined as

$$A=\frac{TP+TN}{TP+FP+TN+FN}$$ (1)

Precision (*P*), Recall (*R*), and the False Positive Rate (*FPR*) are defined as

$$\begin{array}{l} P=\frac{TP}{TP+FP},\\ R=\frac{TP}{TP+FN},\\ FPR=\frac{FP}{FP+TN} \end{array}$$ (2)

The F_β_ score, which is the weighted harmonic mean of *P* and *R*, is expressed as

$$F_{\beta }=\left(1+\beta^{2}\right)\frac{P\times R}{\beta^{2}\times P+R}$$ (3)

where β is a parameter that controls the balance to give more weight to P (β < 1) or R (β > 1).

Due to the imbalance of the dataset classes, it is evident that accuracy (Eq. 1) would not be a proper score for the classification task. Indeed, a classificator that predicts the negative class for all cases would have an accuracy of 86%. It has been shown that, for imbalanced sets, computing precision and recall (Eq. 2) gives a better insight about the classificator’s performance than the Receiver Operating Characteristic curve, a common metric in classification tasks (Saito and Rehmsmeier, 2015). In this context, a low false positive rate is represented by a high precision score, while false negatives are addressed by the recall. In this work, we chose the F_β_ score (Eq. 3) using β = 0.5 to penalize the classifying of molecules that cross the barrier as not crossing, i., e, classification of negative samples as positive samples. Thus, we favor models that have a low number of false positives. A common metric for unbalanced classification problems is the Mathews Correlation Coefficient (MCC); since a recent study discourages its use in unbalanced sets (Zhu, 2020), we decided to use only the F_β_ score due to its direct implementation in penalizing false positives.

The Precision-Recall Curve (PRC) is constructed by plotting *P* in terms of *R* for different probability thresholds. The Average Precision (AP) is a scalar that summarizes the PRC, in the same manner as the area under the curve (AUC) of the receiver operating characteristic (ROC) curve. Strictly, the AP is the area under the PRC.

$$AP=\int_{0}^{1}P\left(R\right)dR$$ (4)

In this work, we approximated this integral by a sum over the precisions at every possible threshold value (*n*) multiplied by the change in *R*, according to

$$AP\approx \sum_{n}P_{n}\left(R_{n}-R_{n-1}\right)$$ (5)

where *R*_*n*_ and *P*_*n*_ are, the recall and precision values at the *n*th threshold value, respectively.

## Results and Discussion

The objective of this study was to provide a ML model capable of classifying compounds either as crossing or not crossing the placental barrier. To this aim, using a dataset of 248 compounds (see Methods), we trained and compared several ML models, searching for optimal low-dimensional sets of descriptors. Considering that the odds of classifying a molecule that crosses the placenta as not crossing must be reduced to a minimum, we chose F_1/2_ as the metric to evaluate performance, thus favoring models that have a low number of FPs; while having a high false rate of predictions is always undesirable, it would be highly risky in this specific case. Due to the high features/samples ratio, we decided to keep the number of descriptors in the final models to a minimum.

### Design of the Feature Selection Protocol

Considering the size of our dataset (248 samples), and the number of calculated features (∼5.4 thousand descriptors), we performed a feature selection process to avoid over-fitting. Initially we considerably reduced the high dimensionality of the feature space by eliminating from the PaDEL set of descriptors variables that did not provide significant information, by eliminating features (specifically, fingerprint counts) that had less than three matches within the molecules of the dataset. This decision was principally based on the trade-off between the number of remaining descriptors (by removing features) and the information loss. After this process, the number of descriptors fell to 760. Needless to say, this procedure is independent of the class labels, and thus can be done before the cross validation split.

To reduce even further the set of 760 features, we used a genetic algorithm (GA) which essentially searches for sets of features with a high F_1/2_ score over a given training set, as described below.

#### Genetic Algorithm

From a training dataset composed of a set of molecules with their corresponding descriptors, the GA generated a population of 1,000 individuals, where each individual was defined as a set of six randomly selected features; we also explored the use of individuals described with nine and 12 features, but did not find any improvement over the use of six features (see *Additional Genetic Algorithm Runs Using Identical Initial Conditions*). Then, each individual was used to train a ML classifier, and subsequently ranked in terms of the obtained F_1/2_ score over the training data. After having this initial population of 1,000 individuals ranked, the following iterative process was carried out: 1) the set of features with the best score of the population (the optimal individual) was assessed; 2) random sets from the top half of the population were selected in pairs and combined until 500 new sets were obtained; with two individuals, a new agent was generated by retrieving the first three features from one individual and the other three features from the other individual; 3) the F_1/2_ score was calculated for each of these 500 generated sets and, independently of the results, these new individuals replaced the bottom half from the past population; 4) the new population of 1,000 features was re-ranked. This iterative process was carried out for 199 iterations, which allowed both the convergence of the method (the top ranked individuals were very similar) and the exploration of the feature space (as explained below).

Within this process, three operations were performed: 1) every time a new set of features was generated (by the combination of two other sets), it was assigned a probability of 0.2 of being mutated. If it was mutated, the new agent would change all its variables with those of the optimal individual, replacing two features with two random ones from the major set; in certain sense, this is a way to explore the “vicinity” of the best scored individual; 2) for each generation we replaced one third of the reproducible population (top half of the population) with new random agents; 3) finally, after 50 iterations, a new initial population of 1,000 individuals was generated and ranked, and the current population was replaced entirely except for the top 10 individuals. The last two operations were performed for the sake of augmenting the exploration of the feature space.

#### Coupling the Genetic Algorithm With the Cross Validation Scheme

**FIGURE 2:**
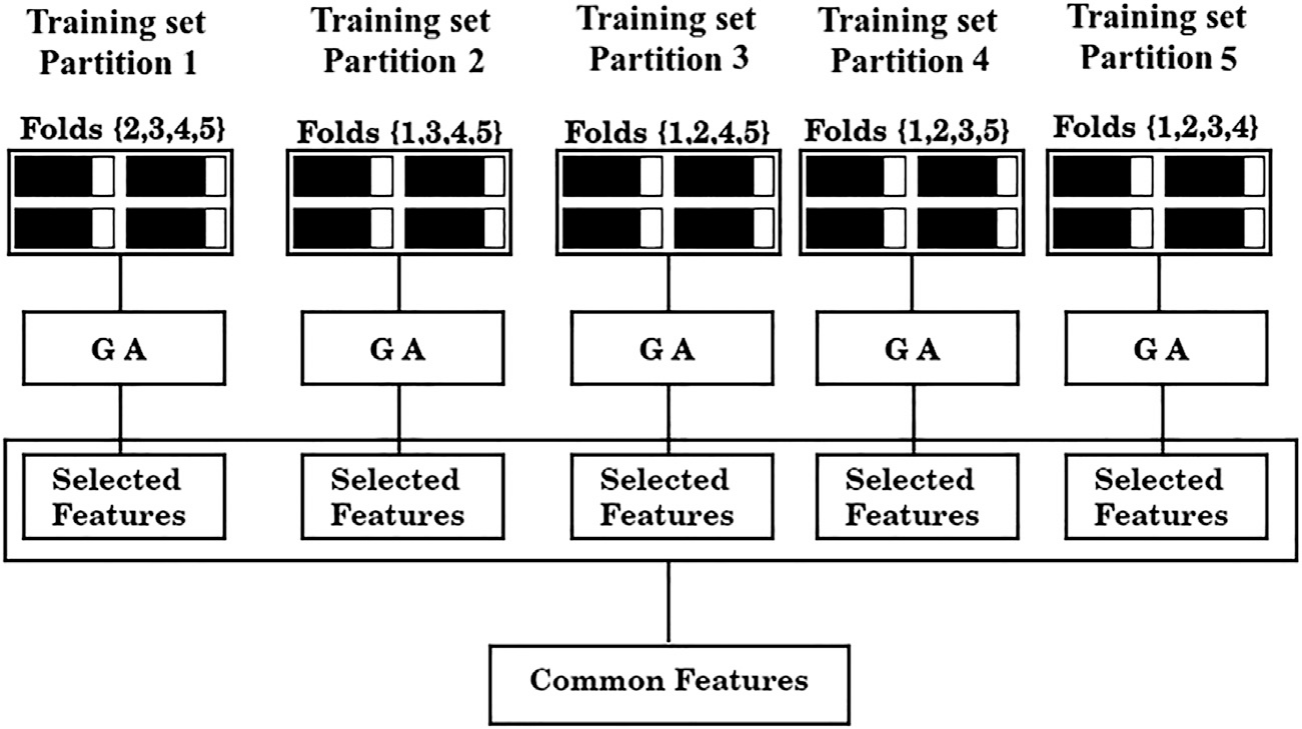
Feature selection scheme. From left to right, training data from partitions 1-5 are fed to a GA. The GA yields a solution for each partition (“Selected features”) and finally, the shared features between those solutions are collected (“Common features”).

**FIGURE 3:**
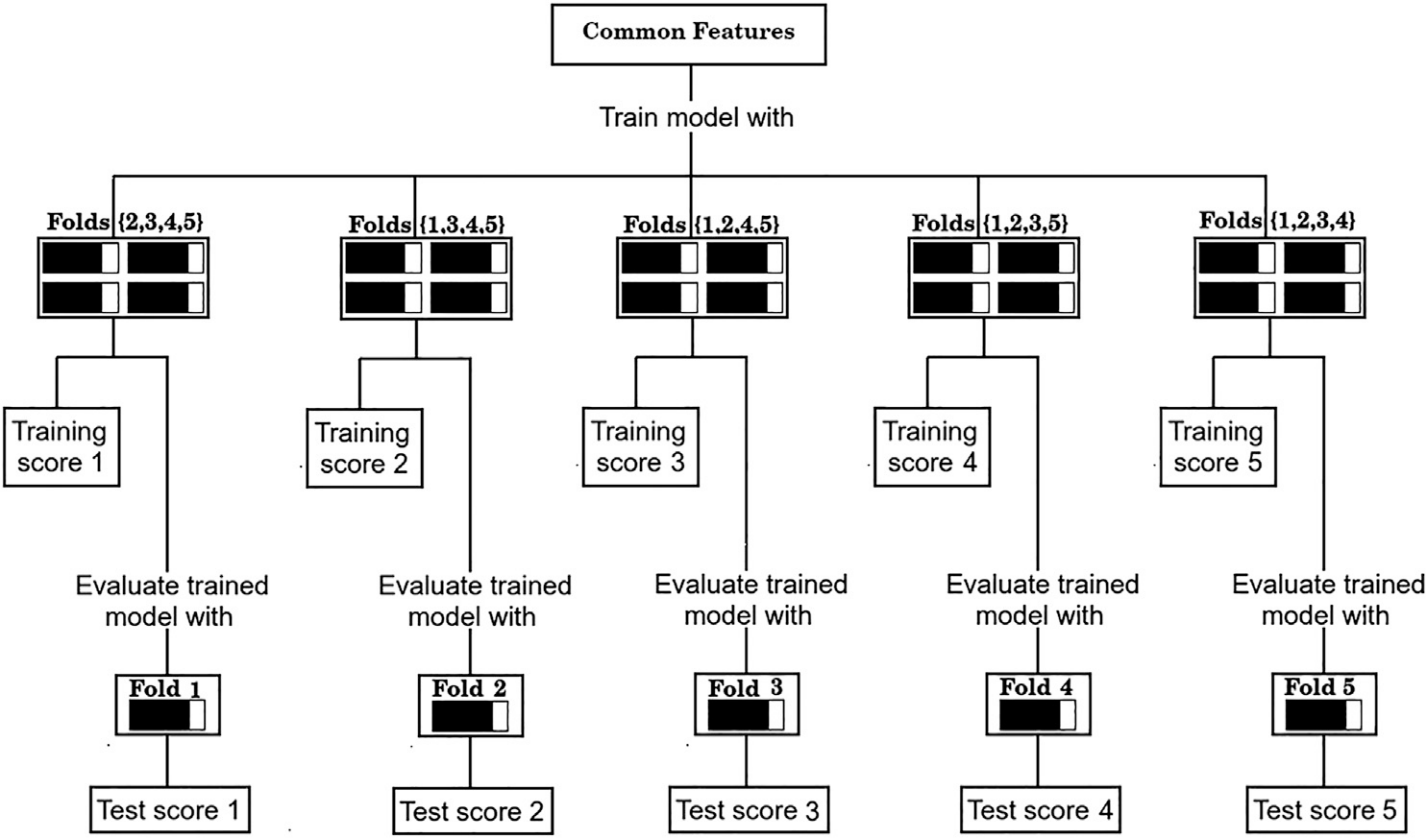
Evaluation procedure of the common sets of features with the 5-fold cross validation scheme.

Following Hastie et al. Hastie et al, (2009), we first split the data according to the cross validation scheme, and then used the feature selection method described above with the training data. Specifically, we proceeded as follows:

1) Divide the total amount of samples into 5 cross-validation folds (*k* = 1, 5) at random as shown in Figure 1 and generate five partitions, where partition *k* corresponds to using fold *k* as a test set, and the remaining four folds as the training set.
2) For each partition, the GA is used to find a set of predictors that exhibits a high value of the F_1/2_ score, calculated only on the training samples.
3) Assess shared features between the optimal sets found in each of the five partitions (common features), as illustrated in Figure 2.
4) Evaluate the performance of the set of common features over the corresponding test sets of each partition, as shown in Figure 3.

As is standard in the use of cross validation schemes, we report the average F_1/2_ scores over the five training sets, and over the five test sets. For simplicity, the process depicted in Figure 2 of finding a set of “Common features” and evaluating it as shown in Figure 3 will be referred from now on as a “run”.

### Selection of the Best Machine Learning Model

Common sets of descriptors were searched for four different ML algorithms: Linear Discriminant Analysis (LDA), Logistic Regression (LR), Random Forest (RF), and Support Vector Machines (SVM). For this task, we ran one GA per model, feeding each algorithm with the same initial population. Before displaying the results corresponding to the four methods, we will illustrate the feature selection protocol with the LDA. In Table 2 we show the best sets of features found in each partition for the LDA model by running a single GA. As mentioned earlier, only the F_1/2_ score over the training set is reported at this stage. These sets of features correspond to the “Selected features” shown in Figure 2.

**TABLE 2:** Best set of features (“Selected features”, see Figure 2) obtained on each partition of the cross validation split based on the training F_1/2_ score for the LDA model.

Partition	Feature 1	Feature 2	Feature 3	Feature 4	Feature 5	Feature 6	F_1/2_ Train
1	KRFPC608	KRFPC841	KRFPC1773	KRFPC3224	KRFPC3591	KRFPC4830	0.87
2	KRFPC413	KRFPC566	KRFPC608	KRFPC1638	KRFPC3399	SubFPC19	0.90
3	KRFPC442	KRFPC557	KRFPC566	KRFPC3400	KRFPC3741	KRFPC3948	0.89
4	KRFPC413	KRFPC566	KRFPC608	KRFPC3139	KRFPC3737	KRFPC4006	0.87
5	KRFPC326	KRFPC566	KRFPC592	KRFPC608	KRFPC3730	KRFPC4830	0.83

Across the five sets of features shown in Table 2, there are four repeated descriptors: KRFPC413 (2 times), KRFPC566 (4 times), KRFPC608 (4 times), and KRFPC4830 (2 times). Although the GA was fed with sets of six features, only these four repeated features constitute the set of “Common features” (cf. Figure 2). Using these four features we trained another LDA model (Figure 3). This model exhibited mean F_1/2_ scores of 0.80 and 0.77 in the training and test sets, respectively (see Table 3). The average F_1/2_ score of 0.77 over the test sets corresponded to average values of *P* and *R* of 0.93 and 0.51, respectively. This represents a very good performance, and *a priori* indicates that it is plausible to select features in this manner.

**TABLE 3:** Repeated features across different partitions for the first run of the GA (“Common features”, see Figures 2, 3) using different ML models. The frequency each feature is repeated within partitions is shown in paretheses. The F_1/2_ Train and F_1/2_ Test columns refer to the average score across the different training folds and test folds, respectively.

Model	Feature 1	Feature 2	Feature 3	Feature 4	Feature 5	Feature 6	F_1/2_ train	F_1/2_ test
RF	KRFPC476 (2)	KRFPC3707 (2)	KRFPC4556 (2)	SubFPC3 (2)	SubFPC301 (2)	MP (2)	1.0	-
LDA	KRFPC413 (2)	KRFPC566 (3)	KRFPC608 (4)	KRFPC4830 (2)	-	-	0.80	0.77
SVM	KRFPC1564 (2)	KRFPC3946 (2)	SubFPC169 (2)		-	-	0.72	-
LR	KRFPC608 (4)	ROTB (2)	-	-	-	-	0.59	0.54

The same process was carried out for the other 3 ML methods. The sets of common features found for each model, as well as the training and test F_1/2_ scores are summarized in Table 3. RF and SVM models were prone to over-fitting, as they achieved a null averaged *R* over the test sets (non-defined F_1/2_ score), and the LR model displayed a significantly poorer performance compared to LDA. We thus continued the analysis with only the LDA model. While different alternatives could be pursued to improve the performance of the other ML models, the aim of this study is to find a robust and accurate model exhibiting high performance.

### Linear Discriminant Analysis Model Analysis

Despite of having promising results with the LDA model (Table 3), at this point it is not yet clear whether the found set of features is robust. Considering the random nature of the GA, we analyzed how the different parameters of the feature selection process could impact on the results. First, we performed five additional runs using the same initial conditions of the GA used for the LDA model shown in Table 3. Then, we focused on three main parameters of the initial conditions of the GA, namely, the number of features used to describe the individuals of the GA, the cross validation split, and the initial population fed to the GA, and performed additional runs maintaining two of the mentioned initial parameters fixed, while varying the third one. In the following results, “run” refers to the finding of a set of common features” and evaluating it (see Figures 2, 3).

#### Additional Genetic Algorithm Runs Using Identical Initial Conditions

Using the same initial population and cross validation split as in the first ML model selection (Table 3), we performed five additional runs of the GA for the LDA model, obtaining another five sets of common features. Results are summarized in Supplementary Table S2. In four of the five runs, KRFPC566 and KRFPC608 belonged to the set of common features and, remarkably, KRPFC3948 was repeated in the five sets. This indicates, that the KRFPC566 and KRFPC608 features, which were found in the first LDA model (Table 3), are retrieved despite of the inherent randomness of the GA, and that the first obtained solution missed an apparently important feature, KRFPC3948.

#### Extending the Size of the Genetic Algorithm Individuals

We performed five runs (see Figures 2, 3) with sets of nine features, and five runs with sets of 12 features. Results are shown in Supplementary Tables S3, S4.

Every set of common features exhibited a low performance in comparison to the LDA model using six features in the GA (Table 3). Over the training data, the highest F_1/2_ score was of 0.60. In the test data, we found one common set for which the model’s performance was of 0.50 (run 3, Supplementary Table S3), and in the rest of the runs, the corresponding models achieved null recall values. This shows that using nine or 12 features in the GA shows no advantage on the performance of the LDA model.

#### Genetic Algorithm Runs Changing the Cross Validation Split

We performed fifteen more runs using the same initial population fed to the GA, but changing the cross validation split three times–five runs per cross validation split. Results are summarized in Supplementary Tables S5-S7. In the first split (Supplementary Table S5) the KRFPC566 feature was found in the common set of features in four of the five runs, which further supports the hypothesis of this descriptor being a key feature. The same applies to the KRFPC3948 descriptor, which was found in three of the five common sets. Two additional features were found: the KRFPC435 descriptor, repeated in two of the five common sets, and the KRFPC4830 descriptor, found in three of the five common sets. Remarkably, one of the common sets found consisted of these four features and obtained an average training F_1/2_ score of 0.81 and an average test F_1/2_ score of 0.78, matching the top performance of the first LDA model (Table 3). Both these sets of common features that display top performances (at least up to this point) share two features, KRFPC566 and KRFPC4830, which indicates that KRFPC4830 may also be a key descriptor.

Although in the second cross validation split (Supplemenatry Table S6) the observed top performance was of 0.54 in the test set, an already encountered descriptor, the KRFPC608 feature, was found repeated in three of the five runs. In the last cross validation split (Supplementary Table S7), the top F_1/2_ score achieved in the test set was of 0.64. The KRFPC435 descriptor was found again in these sets of runs - repeated in three of the five runs-, and also the KRFPC3392 descriptor, found in two of the five runs.

#### Genetic Algorithm Runs Changing the Initial Population

We also performed five extra runs in which the cross validation split was maintained (the same as in the initial run), but changing the initial population fed to the GA. Strictly speaking, this was performed with three different initial populations, totalizing fifteen extra runs. Results are shown in Supplementary Tables S8-S10. The KRFPC566 descriptor was found to be repeated in eight of the fifteen runs, thus clearly indicating that this feature is indeed important to achieve a high F_1/2_ score with the LDA model. For the first change in the initial population, i.e., the first five runs, the KRFPC3948 descriptor was found in four of the five common sets. Although it was not found repeated in the remaining ten runs, it must be taken into consideration that this descriptor had already been found previously in a high performance set (Supplementary Table S5). The KRFPC435 descriptor shows a similar behavior, which was found earlier along with the KRFPC3948 descriptor (Supplementary Table S5): the results of the second change in the initial population (Supplementary Table S9) show that the KRFPC435 descriptor is repeated in two of five common sets. Other descriptors were also found repeated within the common sets, but at this point we considered them as irrelevant since they did not show up in any of the previous results, specifically, the KRFPC3899 and the KRFPC669 descriptors. Similar to the change in the cross validation split, where one particular change led to a top performing model (Supplementary Table S5), and the two other led to models with a poor performance (Supplementary Tables S6, S7), the same happens with the change in the initial population: The performances shown in Supplementary Tables S9-S10 are low in comparison to previous results. Nonetheless, a particular run shown in Supplemenatary Table S8 presents the best performance so far. This set of features included the KRFPC435, KRFPC566 and KRFPC3948 descriptors, along with KRFPC3399 and KRFPC3899. The first three descriptors were already included in a high performance model (Run 1 from Supplementary Table S5). Presumably, the last two descriptors are only complementary features (to the first three) related to the change of the initial population.

### Final Linear Discriminant Analysis Model

From the previous results (Table 3; Supplementary Table S4-S10), we show in Table 4 the three sets with the best performances. These correspond to the LDA run from Table 3, run 1 from Supplementary Table S5, and run 1 from Supplemenatary Table S8.

**TABLE 4:** Best set of common features found in the complete set of runs.

	Feature 1	Feature 2	Feature 3	Feature 4	Feature 5	F_1/2_ Train	F_1/2_ Test
LDA (Table 3)	KRFPC435	KRFPC566	KRFPC3948	KRFPC4830	-	0.81	0.78
Run 1 (Supplementary Table S5)	KRFPC413	KRFPC566	KRFPC608	KRFPC4830	-	0.80	0.77
Run 1 (Supplementary Table S6)	KRFPC435	KRFPC566	KRFPC3948	KRFPC3399	KRFPC3899	0.80	0.78

To compare these sets of features, we assessed, feature by feature, in which of the previous runs (Table 3; Supplementary Table S4-S10), each descriptor was present. For each table, we distinguish three cases: 1) the feature was not present in any of the runs of the corresponding table; 2) the feature was present only in one run; 3) the feature was present in more than one run. To quantify the appearance of features across different runs we assigned a partial score to each of the cases described before, being 0 for 1), ½ for 2) and 1 for 3). Taking into account that in Table 3 there is only one LDA run, the sum of partial scores ranges from 0 to 7.5. The feature importance was defined, for each feature, as the sum of partial scores normalized by 7.5, so that the ranking goes between 0 and 1. This information is summarized in Table 5, which allows the visualization of which features are repeated even when the initial conditions of the selection process were changed consistently, like KRFPC566, and which features appear to be dependent on a particular condition of the same process, such as KRFPC608, which appears only in Table 3; Supplementary Table S4, corresponding to the exact same conditions. The comparison between the sets of features presented in Table 4 in terms of the feature importance of each descriptor supports the fact that the set of features composed by KRFPC435, KRFPC566, KRFPC3948 and KRFPC4830 descriptors (Table 4, highlighted in bold) is the most robust. Intuitively, the selected features were the ones which were found more often in the different runs in which the initial conditions of the optimization process were changed. Given a compound, these four features describe the number of times a specific SMARTS pattern is repeated along the molecular structure. The SMARTS associated with each descriptor are shown in Table 6.

**TABLE 5:** Analysis of the repeated features over different runs. The column headers display each of the features that appear in Table 4. The rows contain information on whether these features were present or not in each of the performed runs: XX indicates that the feature was repeated across common sets of features, and X indicates the presence of the feature in only one common set.

	KRFPC435	KRFPC566	KRFPC3948	KRFPC4830	KRFPC413	KRFPC608	KRFPC3399	KRFPC3899
Initial run (Table 3)	-	X	-	X	X	X	-	-
Extra five runs (Supplementary Table S2)	-	XX	XX	X	-	XX	-	-
Change in cross validation split (Supplementary Table S5)	XX	XX	XX	X	-	-	-	X
Change in cross validation split (Supplemenatry Table S6)	-	X	-	-	-	XX	-	-
Change in cross validation split (Supplementary Table S7)	XX	-	-	X	-	X	-	-
Change initial population (Supplemenatary Table S8)	X	XX	XX	-	-	-	X	XX
Change initial population (Supplementary Table S9)	XX	XX	X	X	X	-	-	-
Change initial population (Supplementary Table S10)	X	XX	-	X	-	-	-	-
Feature importance	0.53	0.8	0.47	0.4	0.13	0.4	0.07	0.2

**TABLE 6:** SMARTS patterns associated with set of descriptors of the final LDA model. R represents any atom other than Hydrogen.

KlekotahRoth Fingerprint Count	SMARTS	Molecular Structure
KRFPC435	[#6]-[#7](-[!#1])-[#6]-[#6]-[!#1]	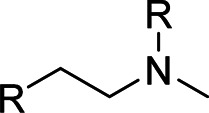
KRFPC566	[!#1]-[#6]-[#6]-1 = [#6]-[#6] = [#6]-[#6] = [#6]-1	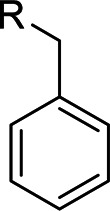
KRFPC3948	[#6]-[#7]-[#6]-[#6]-[#8]	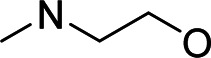
KRFPC4830	[#8]-[#6]-[#6]-[#8]-[#6] = [#8]	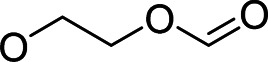

### Analysis of Misclassified Molecules Within the Final Model

**FIGURE 4:**
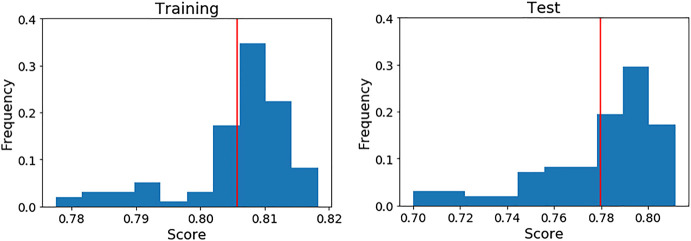
Performance of the final LDA model over 100 different five cross validation splits. The red line indicates the mean score on each case. Left: Training data. Right: Test data.

It is important to bear in mind that the scores of the final model shown in Table 4 (highlighted in bold) were achieved over a particular cross validation split. To ensure that the score achieved with these features was not highly dependent on that particular split, we generated 100 different splits and evaluated the model’s scores on each one (see Figure 4).

As can be seen, the mean scores are close to the achieved values in the initial split. For this reason, we present the full performance over the test set on each fold (Table 7) using that initial cross validation split. TP, FP, FN and TN are also computed to show exactly how the LDA model is classifying the compounds.

**TABLE 7:** Results using the best set of features (KRFPC435, KRFPC566, KRFPC3948 and KRFPC4830) on each partition over the corresponding test sets.

Partition	F_1/2_ Test	Precision	Recall	AP	TP	FP	FN	TN
1	0.79	1.0	0.43	0.54	3	0	4	43
2	0.74	0.8	0.57	0.63	4	1	3	42
3	0.66	1.0	0.29	0.40	2	0	5	43
4	0.79	1.0	0.43	0.73	3	0	4	42
5	0.93	1.0	0.71	0.76	5	0	2	42
**Average**	**0.78**	**0.96**	**0.49**	**0.61**	-	-	-	-

Remarkably, there is only one negative sample misclassified, thus achieving the most important objective sought for this classifier. A great balance is observed between the total number of TPs (17) and FNs (18), which in conjunction with the correct classification of the negative class, gives an overall excellent performance. To use this model prospectively, given a new set of molecules, the final model would have to be trained with our entire dataset of 248 compounds. For the new compounds, we would only calculate the KRFPC435, KRFPC566, KRFPC3948 and KRFPC4830 descriptors, and placental transfer would be predicted by inputting the new set of molecules to the ML model.

#### False Positive Case

The only FP observed in the test sets corresponds to Tubocuraine (CID = 6,000), which belongs to fold 2. As a matter of fact, when this compound is used to train the LDA model, and this trained model is used to make predictions over the corresponding training set (partitions 1, 3, 4, and 5), this compound is also miss-classified, so this is the only compound belonging to the negative class that is misclassified both in the training and test sets.

Compound 6,000 is described with the following descriptors: KRFPC435 = 4, KRFPC566 = 0, KRFPC3948 = 0 and KRFPC4830 = 0. Similar molecules from the database in terms of these four features, i.e., compounds with KRFPC435 > 0 and the rest of the descriptors equal to zero, are listed in Table 8, together with their actual placental transfer class (C or NC).

**TABLE 8:** Compounds from the database similar to Tubocuraine (CID = 6,000, in bold) in terms of the four descriptors of the final model. The“Cross” column contains the actual placental transfer class (C or NC).

CID	Name	KRFPC435	KRFPC566	KRFPC3948	KRFPC4830	Cross
47,320	Atracurium Besilate	8	0	0	0	NC
21,233	Dimethyl-Tubocurarine	6	0	0	0	NC
**6,000**	**Tubocuraine**	**4**	**0**	**0**	**0**	**C**
5,750	Pethidine (Meperidine)	2	0	0	0	C
4,062	Mepivacaine	1	0	0	0	C
43,708	Cefotiam	1	0	0	0	C
89,594	Nicotine	1	0	0	0	C
5288826	Morphine	1	0	0	0	C
2,801	Clomipramine	1	0	0	0	C

Taking into account that compound 6,000 is the only FP in the training and test sets, and considering that the rest of the compounds that cross the placenta shown in Table 8 are correctly classified whether they were in the training or the test set, it is reasonable to suppose that in the case of having null values in the KRFPC566, KRFPC3948 and KRFPC4830 descriptors, classes are distinguished based on a threshold in the KRFPC435 value: compounds with KRFPC435 ≤ 4 cross the placenta, while compounds with a KRFPC435 > 5 do not cross it.

To assess which threshold our LDA model–trained with the 248 compounds has learned, we inputted several artificial samples with KRFPC435 values ranging from one to nine and the rest of the descriptors with values equal to zero. We confirmed that compounds are classified as not crossing the placental barrier with KRFPC435 ≥ 4, which explains why compound 6,000 is misclassified.

#### False Negative Cases

From Table 7 18 FNs were identified in the test sets. Inspecting the representation of the database in terms of the optimal descriptors, we found that the majority of the compounds that cross the placenta were described by null values in the four descriptors (161 compounds), or had only KRFPC3948 > 0 (31 compounds). Of the 18 FNs, we found 17 compounds that had one of the representations described before (corresponding to compounds crossing the placenta): 12 compounds had all the four values equal to zero and five compounds had only KRFPC3948 > 0. The remaining FN corresponds to compound 441243. Surprisingly, there is another compound (CID = 5362440) with the same representation (KRFPC435 = 0, KRFPC566 = 1, KRFPC4830 = 0 and KRFPC3948 = 3) that does not cross the placenta and which is not misclassified. As these two compounds belong to different folds, and effectively checking that there is no compound that crosses the placenta with this exact representation, we assume that the misclassification of compound 441243 is directly related to the cross validation split. Unlike the other 17 FNs, miss-classifications like compound 441243 could be avoided in prospective applications (by the use of both 441243 and 5362440 compounds in the training set).

It is clear that the majority of FNs arise due to there being compounds belonging to different classes (C-NC) with the same representation. In fact, the positive samples that were correctly classified in the test sets (Table 7) presented clear distinctions in their representations with respect to negative samples. This indicates that to reduce the amount of FNs, at least one more feature should be incorporated. As simple as this may sound, directly incorporating new descriptors to this particular set of features would introduce a bias into the solution [because the relationship between descriptors and classes (C-NC) is already known for the entire dataset], and would finally result in an overfitted model. Although this is a limitation of our method, taking into account that the amount of TPs is at an acceptable level, and that the main goal of having low amounts of false positives was fulfilled by the use of the F_1/2_ score, we consider performing further GA searches or modifying any of the feature selection protocol parameters unnecessary.

## Conclusion

The study of chemical transfer across the placental membrane from the mother to the fetus is of the utmost importance due to its importance to drug safety, especially in a time when drug prescription during pregnancy is common. Taking into account that *in vivo* data cannot be obtained for ethical reasons, the main difficulty arises from the fact that *in vitro* and *ex vivo* methods cannot directly predict *in vivo* outcomes. In this scenario, the use of *in silico* approaches to complement *ex vivo* and *in vitro* models constitutes an interesting strategy to tackle this challenge.

Although QSAR models have been developed, the datasets used for developing these models were rather small (<100 compounds), and the models validated only on small test sets (<20 compounds). In this study, a database of 248 compounds was compiled, and although this still remains a small dataset, to our knowledge it is the largest reported so far. Also, unlike those studies, which predicted either the CI or the F/M ratio, we treated the placental transfer as a binary classification problem (cross/not cross) rather than as a regression task for a continuous variable.

The results shown in this work support the use of our feature selection protocol, which involves the implementation of a GA that maximizes the F_1/2_ score in conjunction with a five cross validation scheme. The final LDA model displayed key characteristics that are desirable for a ML classificator in this context: 1) it relies on a set of only four features to discriminate between classes; 2) it correctly classifies the majority of both classes; 3) most importantly, the number of molecules that cross the placenta predicted by the LDA model as not crossing was very low.

One limitation of our ML model is that it was trained with a low amount of data (*N* ∼ 250). Strictly speaking, this limitation is not intrinsic to the model itself, but related to our knowledge of placental transfer itself, since there is scarce reliable information publicly available.

As we highlighted before, despite having a low amount of positive (non-crossing) samples, the fact of having only one false positive along the test sets is remarkable. Considering also that a significant number of molecules within the positive class was correctly classified in the test sets (approximately, half of the corresponding positive samples), this supports the incorporation of a ML predictor of placental membrane crossing in a drug discovery campaign.

## Data Availability

The original contributions presented in the study are included in the article/Supplementary Material, further inquiries can be directed to the corresponding author.
