# Supplementary material for: A Machine Learning Model to Predict Drug Transfer Across the Human Placenta Barrier
Source: Front Chem. 2021 Jul 20;9:714678. doi: 10.3389/fchem.2021.714678 (PMC8329444; doi:10.3389/fchem.2021.714678)
Supplement: Supplementary file 1 [file DataSheet1.PDF]

Supplementary Information

A machine learning model to predict drug transfer across the human placenta barrier

Juan I. Di Filippo[1,2,3], Mariela Bollini[4], Claudio N. Cavasotto[1,2,3,*]

[1]Computational Drug Design and Biomedical Informatics Laboratory, Instituto de Investigaciones en Medicina Traslacional (IIMT), CONICET-Universidad Austral, Pilar, Buenos Aires, Argentina

[2]Facultad de Ciencias Biomédicas, and Facultad de Ingeniería, Universidad Austral, Pilar, Buenos Aires, Argentina

[3]Austral Institute for Applied Artificial Intelligence, Universidad Austral, Pilar, Buenos Aires, Argentina

[4]Centro de Investigaciones en BioNanociencias (CIBION), Consejo Nacional de Investigaciones Científicas y Técnicas (CONICET), Ciudad de Buenos Aires, Argentina

*** Correspondence:**
Corresponding Author
CCavasotto@austral.edu.ar; cnc@cavasotto-lab.net

Table of Contents

Table S1 .... S2-S13

Tables S2-S4 ....S14

Tables S5-S6 ....S15

Tables S7-S9 ....S16

Table S10 ....S17

References..................................... S18-S22

Table S1. Observed experimental values of clearence index (CI) and/or fetus:mother ratio (F/M) of our compound database. Whenever available, the experimental cross/no-cross [C/NC(exp.)] classification from the literature is also reported. The binary classification of cross/no cross (Cross column) used to train and test our models was based on the criteria explained in Methods (section 2.1). We show in column Criterion which element was used to classify each compound [CI, F/M, or C/NC(exp)].

No	CID	Drug (commercial)	Cross	CI[a]	Ref.	F/M	Ref.	C/NC (exp)	Ref.	Criterion
1	3121	Valproic acid (Valproate)	Yes	0.95	(Giaginis et al., 2009)	1.51	(Takaku et al., 2015)			F/M
2	1983	Acetaminophen (Paracetamol)	Yes			1.34	(Pacifici and Nottoli, 1995)			F/M
3	33613	Amoxicillin	Yes			0.84	(Pacifici and Nottoli, 1995)			F/M
4	6087	Methicillin	Yes			0.81	(Pacifici and Nottoli, 1995)			F/M
5	2369	Betaxolol	Yes			0.93	(Morselli et al., 1990)			F/M
6	135398513	Acyclovir	Yes	0.17	(Giaginis et al., 2009)	0.84	(Gilstrap et al., 1994; Pacifici and Nottoli, 1995)			F/M
7	37768	Amikacin	Yes			0.67	(Pacifici and Nottoli, 1995)			F/M
8	2519	Caffeine	Yes			1.00	(Pacifici and Nottoli, 1995; Mose et al., 2008)			F/M
9	2153	Theophylline	Yes	0.80	(Giaginis et al., 2009)			C	(Pacifici and Nottoli, 1995; Grosso et al., 2006)	C/NC(exp)
10	6249	Ampicillin	Yes			0.75[b]	(Chow and Jewesson, 1985; Pacifici and Nottoli, 1995)			F/M
11	5284519	Azlocillin	Yes			1.00	(Pacifici and Nottoli, 1995)			F/M
12	9570757	Cefmenoxime	Yes			0.30	(Pacifici and Nottoli, 1995)			F/M
13	441199	Cefoxitin	Yes			0.60	(Pacifici and Nottoli, 1995)			F/M
14	38103	Cephradine	Yes					C	(Pacifici and Nottoli, 1995)	C/NC(exp)
15	5773	Cephaloridine	Yes			1.15[b]	(Ward, 1996)			F/M
16	43708	Cefotiam	Yes					C	(Pacifici, 2006)	C/NC(exp)

No	CID	Drug (commercial)	Cross	CI[a]	Ref.	F/M	Ref.	C/NC (exp)	Ref.	Criterion
17	6533629	Ceftizoxime	Yes	0.12	(Giaginis et al., 2009)	>1.0	(Pacifici, 2006)			F/M
18	5479530	Ceftriaxone	Yes					C	(Cho et al., 1988)	C/NC(exp)
19	3016	Diazepam	Yes			1.26	(Takaku et al., 2015)			F/M
20	2809	Clorazepate	Yes			1.00	(Rey et al., 1979)			F/M
21	3958	Lorazepam	Yes			0.50	(Kanto, 1982)			F/M
22	4192	Midazolam	Yes			0.74	(Takaku et al., 2015)			F/M
23	4506	Nitrazepam	Yes			>0.6	(Kanto, 1982)			F/M
24	4616	Oxazepam	Yes			>0.6	(Kanto, 1982)			F/M
25	40391	Pinazepam	Yes			0.64	(Pacifici et al., 1984)			F/M
26	2719	Chloroquine	Yes			0.93	(Takaku et al., 2015)			F/M
27	3715	Indomethacin	Yes	0.72	(Giaginis et al., 2009)	0.98	(Takaku et al., 2015)			F/M
28	5253	Sotalol	Yes			0.50	(Pacifici and Nottoli, 1995; Takaku et al., 2015)			F/M
29	38853	Methyldopa	Yes			1.10[b]	(Cho et al., 1988)			F/M
30	4763	Phenobarbitone (Phenobarbital)	Yes	0.52	(Giaginis et al., 2009)			C	(Cho et al., 1988)	C/NC(exp)
31	1775	Phenytoin	Yes	0.52	(Giaginis et al., 2009)	1.00	(Cho et al., 1988)	C	(Cho et al., 1988)	C/NC(exp)
32	5288826	Morphine	Yes	0.63	(Giaginis et al., 2009)	0.73	(Kopecky et al., 1999)	C	(Cho et al., 1988)	F/M
33	5193	Secobarbital (Quinalbarbitone)	Yes					C	(Root et al., 1961; Cho et al., 1988)	C/NC(exp)
34	3032285	Thiamylal (Tiamil)	Yes					C	(Cho et al., 1988)	C/NC(exp)
35	4906	Prilocaine	Yes			1.00	(Root et al., 1961)			F/M
36	4116	Methoxyflurane	Yes			0.60[b]	(Cho et al., 1988)			F/M
37	3821	Ketamine	Yes			0.60[b]	(Cho et al., 1988)			F/M
38	4943	Propofol	Yes	0.51	(Giaginis et al., 2009)	>0.65	(Cho et al., 1988)			F/M

No	CID	Drug (commercial)	Cross	CI[a]	Ref.	F/M	Ref.	C/NC (exp)	Ref.	Criterion
39	948	Nitrous Oxide	Yes			0.80	(Littleford, 2004)			F/M
40	35370	Zidovudine (AZT)	Yes	0.29	(Giaginis et al., 2009)	0.92	(McCormack and Best, 2014)			F/M
41	18283	Stavudine (d4T)	Yes	0.24	(Giaginis et al., 2009)	0.93	(Takaku et al., 2015)			F/M
42	60825	Lamivudine (3TC)	Yes	0.23	(Giaginis et al., 2009)	1.31	(Takaku et al., 2015)			F/M
43	135398739	Didanosine	Yes	0.31	(Giaginis et al., 2009)	0.38	(McCormack and Best, 2014)			F/M
44	4463	Nevirapine	Yes			0.67	(McCormack and Best, 2014)			F/M
45	24066	Zalcitabine	Yes	0.22	(Giaginis et al., 2009)	1.00	(Allegaert and Van Den Anker, 2017)			F/M
46	1978	Acebutolol	Yes			0.79	(Takaku et al., 2015)			F/M
47	2249	Atenolol	Yes			1.30[b]	(Cho et al., 1988)			F/M
48	2803	Clonidine	Yes			0.89	(Takaku et al., 2015)			F/M
49	3784	Isradipine	Yes			0.40	(Cho et al., 1988)			F/M
50	3676	Lignocaine (Lidocaine)	Yes	0.91	(Giaginis et al., 2009)	0.55	(Pacifici and Nottoli, 1995; Takaku et al., 2015)			F/M
51	4062	Mepivacaine	Yes			0.69	(Brown et al., 1975)			F/M
52	6032	Kanamycin	Yes			0.50	(Pacifici and Nottoli, 1995)			F/M
53	441278	Pentazocine	Yes			0.60	(Pacifici and Nottoli, 1995)			F/M
54	5750	Pethidine (Meperidine)	Yes			0.81	(Tomson et al., 1982; Pacifici and Nottoli, 1995)			F/M
55	2554	Carbamazepine	Yes			0.79	(Bank et al., 2017)			F/M
56	4909	Primidone	Yes			0.99[b]	(Pacifici and Nottoli, 1995)			F/M
57	3639	Hydrochlorothiazide	Yes			0.50[b]	(Pacifici and Nottoli, 1995)			F/M
58	2724385	Digoxin	Yes			0.50	(Pacifici and Nottoli, 1995)			F/M
59	5403	Terbutaline	Yes			1.00	(Pacifici and Nottoli, 1995)			F/M
60	174174	Atropine	Yes					C	(Pacifici and Nottoli, 1995)	C/NC(exp)

No	CID	Drug (commercial)	Cross	CI[a]	Ref.	F/M	Ref.	C/NC (exp)	Ref.	Criterion
61	2206	Antipyrine	Yes	0.98	(Sudhakaran et al., 2005)			C	(Hewitt et al., 2007)	C/NC(exp)
62	446987	Fosfomycin	Yes			>0.50	(Pacifici and Nottoli, 1995)			F/M
63	441140	Griseofulvin	Yes			0.80	(Pacifici and Nottoli, 1995)			F/M
64	5339	Sulfasalazine	Yes			0.58	(Pacifici and Nottoli, 1995)			F/M
65	27200	Thiamphenicol	Yes					C	(Pacifici and Nottoli, 1995; Pacifici, 2006)	C/NC(exp)
66	4737	Pentobarbitone (Pentobarbital)	Yes			0.80	(Pacifici and Nottoli, 1995)			F/M
67	3763	Isoflurane	Yes			0.27	(Brown et al., 1975)			F/M
68	9034	Methohexital	Yes			0.87	(Herman et al., 2000)			F/M
69	7239	1.2-Dichlorobenzene	Yes	0.98	(Hewitt et al., 2007)					CI
70	3345	Fentanyl	Yes			0.37	(Loftus et al., 1995)			F/M
71	702	Ethanol	Yes	1.07	(Giaginis et al., 2009)					CI
72	89594	Nicotine	Yes	0.93	(Giaginis et al., 2009)					CI
73	4993	Pyrimethamine	Yes	1.00	(Giaginis et al., 2009)					CI
74	3000715	Thiopentone (Thiopental)	Yes	0.95	(Giaginis et al., 2009)					CI
75	15130	L-Alphacetylmethadol	Yes	0.95	(Giaginis et al., 2009)					CI
76	40692	Mefloquine	Yes	1.57	(Giaginis et al., 2009)					CI
77	64139	Efavirenz	Yes			0.49	(Cressey et al., 2012)	C	(Weiner, 2019)	F/M
78	5426	Thalidomide	Yes					C	(Weiner, 2019)	C/NC(exp)
79	5362124	Benazepril	Yes					C	(Weiner, 2019)	C/NC(exp)
80	55891	Fosinopril	Yes					C	(Weiner, 2019)	C/NC(exp)
81	5362119	Lisinopril	Yes					C	(Weiner, 2019)	C/NC(exp)
82	91270	Moexipril	Yes					C	(Mosby, 2017)	C/NC(exp)

No	CID	Drug (commercial)	Cross	CI[a]	Ref.	F/M	Ref.	C/NC (exp)	Ref.	Criterion
83	54892	Quinapril	Yes					C	(Weiner, 2019)	C/NC(exp)
84	5362129	Ramipril	Yes					C	(Weiner, 2019)	C/NC(exp)
85	44093	Captopril	Yes					C	(Mosby, 2017)	C/NC(exp)
86	5388962	Enalapril	Yes					C	(Weiner, 2019)	C/NC(exp)
87	5284513	Acitretin	Yes					C	(Pilkington and Brogden, 1992)	C/NC(exp)
88	5282379	Isotretinoin	Yes					C	(Weiner, 2019)	C/NC(exp)
89	54676038	Dicoumarol	Yes			0.79	(Pacifici and Nottoli, 1995)			F/M
90	54676537	Acenocoumarol	Yes					C	(Weiner, 2019)	C/NC(exp)
91	54678486	Warfarin	Yes					C	(Weiner, 2019)	C/NC(exp)
92	53232	Lovastatin	Yes					C	(Weiner, 2019)	C/NC(exp)
93	446155	Fluvastatin	Yes					C	(Weiner, 2019)	C/NC(exp)
94	54454	Simvastatin	Yes					C	(Weiner, 2019)	C/NC(exp)
95	60823	Atorvastatin	Yes					C	(Weiner, 2019)	C/NC(exp)
96	446156	Cerivastatin	Yes					C	(Weiner, 2019)	C/NC(exp)
97	1990	Acetohydroxamic acid	Yes					C	(Thakur et al., 2011)	C/NC(exp)
98	57363	Finasteride	Yes					C	(Thakur et al., 2011)	C/NC(exp)
99	448537	Diethylstilbestrol	Yes					C	(Weiner, 2019)	C/NC(exp)
100	1349907	Methimazole	Yes					C	(Weiner, 2019)	C/NC(exp)
101	444795	Tretinoin (Retinoic acid)	Yes					C	(Weiner, 2019)	C/NC(exp)
102	6013	Testoterone	Yes					C	(Hollier et al., 2014)	C/NC(exp)
103	25249	Stanozolol	Yes					C	(Caballero et al., 2014)	C/NC(exp)
104	3037705	Metenolone	Yes					C	(Barceloux, 2012)	C/NC(exp)
105	9904	Nandrolone	Yes					C	(Barceloux, 2012)	C/NC(exp)

No	CID	Drug (commercial)	Cross	CI[a]	Ref.	F/M	Ref.	C/NC (exp)	Ref.	Criterion
106	54675776	Tetracycline	Yes					C	(Weiner, 2019)	C/NC(exp)
107	54675779	Oxytetracycline	Yes			0.55[b]	(Pacifici and Nottoli, 1995)			F/M
108	60846	Valsartan	Yes					C	(Weiner, 2019)	C/NC(exp)
109	3961	Losartan	Yes					C	(Weiner, 2019)	C/NC(exp)
110	5743	Dexamethasone	Yes	0.37	(Giaginis et al., 2009)			C	(Weiner, 2019)	C/NC(exp)
111	249266	2-Chlorobiphenyl	Yes					C	(Örberg, 1977; Ando et al., 1985)	C/NC(exp)
112	16322	3-Chlorobiphenyl	Yes					C	(Örberg, 1977; Ando et al., 1985)	C/NC(exp)
113	16323	4-Chlorobiphenyl	Yes					C	(Örberg, 1977; Ando et al., 1985)	C/NC(exp)
114	3393	Flurazepam	Yes					C	(Weiner, 2019)	C/NC(exp)
115	5556	Triazolam	Yes					C	(Baker et al., 1981)	C/NC(exp)
116	5852	Penicillamine	Yes					C	(Weiner, 2019)	C/NC(exp)
117	5576	Trimethadione	Yes					C	(Weiner, 2019)	C/NC(exp)
118	19649	Streptomycin	Yes					C	(Ormerod, 2001)	C/NC(exp)
119	441300	Abacavir	Yes	0.47	(Giaginis et al., 2009)	1.02	(McCormack and Best, 2014)			F/M
120	51263	Alfentanil	Yes	0.75	(Giaginis et al., 2009)	0.35	(Pacifici and Nottoli, 1995)			F/M
121	644073	Buprenorphine	Yes	0.29	(Giaginis et al., 2009)	0.35	(Takaku et al., 2015)			F/M
122	8612	Chloroprocaine	Yes	0.83	(Giaginis et al., 2009)					CI
123	5280980	Clavulanic acid	Yes	0.06	(Giaginis et al., 2009)	0.56	(Root et al., 1961; Fortunato et al., 1992)			F/M
124	222786	Cortisone	Yes	0.74	(Giaginis et al., 2009)			C	(Freyer, 2009)	C/NC(exp)
125	3033	Diclofenac	Yes	0.79	(Giaginis et al., 2009)	~1.0	(Weiner, 2019)			F/M
126	3637	Hydralazine	Yes	0.61	(Giaginis et al., 2009)	>1.0	(Weiner, 2019)			F/M

No	CID	Drug (commercial)	Cross	CI[a]	Ref.	F/M	Ref.	C/NC (exp)	Ref.	Criterion
127	4168	Metoclopramide	Yes	0.40	(Giaginis et al., 2009)			C	(Pacifici and Nottoli, 1995)	C/NC(exp)
128	4095	Methadone	Yes	0.83	(Giaginis et al., 2009)			C	(Weiner, 2019)	C/NC(exp)
129	5284596	Naloxone	Yes	0.64	(Giaginis et al., 2009)	0.50	(Takaku et al., 2015)			F/M
130	493570	Riboflavin	Yes	0.69	(Dancis et al., 1985)			C	(Weiner, 2019)	C/NC(exp)
131	3825	Ketoprofen	Yes	0.39	(Giaginis et al., 2009)			C	(Weiner, 2019)	C/NC(exp)
132	1548887	Sulindac	Yes	0.47	(Giaginis et al., 2009)	0.40	(Weiner, 2019)			F/M
133	5352624	Sulindac Sulfide	Yes	0.81	(Giaginis et al., 2009)	~0.40	(Weiner, 2019)			F/M
134	5546	Triamterene	Yes	0.85	(Giaginis et al., 2009)	~1.0	(Weiner, 2019)			F/M
135	5310993	Acipimox	Yes	0.80	(Ghabrial et al., 1991)					CI
136	171548	Biotin (Vitamin H)	Yes	0.35	(Giaginis et al., 2009)			C	(Baker et al., 1981)	C/NC(exp)
137	60848	Atevirdine	Yes	0.72	(Roberts et al., 1995)	0.40[b]	(Roberts et al., 1995)			F/M
138	6106	L-Leucine	Yes	0.76	(Giaginis et al., 2009) (Paolini et al., 2001)			C	(Cetin et al., 1995)	C/NC
139	644006	Cocaethylene	Yes	0.78	(Giaginis et al., 2009)			C	(Abman and Steven, 2017)	C/NC(exp)
140	446220	Cocaine	Yes	0.88	(Giaginis et al., 2009)			C	(Abman and Steven, 2017)	C/NC(exp)
141	33572	Ritodrine	Yes	0.10	(Giaginis et al., 2009)	0.49[b]	(Pacifici and Nottoli, 1995)	C	(Fujimoto et al., 1991)	C/NC(exp)
142	1054	Pyridoxine	Yes	0.56	(Giaginis et al., 2009)			C	(Weiner, 2019)	C/NC(exp)
143	4685	1.4-Dichlorobenzene	Yes	0.98	(Giaginis et al., 2009)					CI
144	5755	Prednisolone	Yes	0.38	(Giaginis et al., 2009)			C	(Weiner, 2019)	C/NC(exp)
145	37248	PCB-52	Yes	0.74	(Giaginis et al., 2009)			C	(Lancz et al., 2015)	C/NC(exp)
146	2730	Chlorpyrifos	Yes			0.98	(Takaku et al., 2015)			F/M
147	3017	Diazinon	Yes			1.00	(Takaku et al., 2015)			F/M
148	7430	Dicloran	Yes			1.07	(Takaku et al., 2015)			F/M
149	3356	Flecainide	Yes			0.63	(Takaku et al., 2015)			F/M

No	CID	Drug (commercial)	Cross	CI[a]	Ref.	F/M	Ref.	C/NC (exp)	Ref.	Criterion
150	3767	Isoniazid	Yes			0.62	(Takaku et al., 2015)			F/M
151	4171	Metoprolol	Yes			1.00	(Takaku et al., 2015)			F/M
152	37497	Etidocaine	Yes			0.34	(Pacifici and Nottoli, 1995; Takaku et al., 2015)			F/M
153	4173	Metronidazol	Yes			1.00	(Takaku et al., 2015)			F/M
154	288	Carnitine	Yes			1.29	(Takaku et al., 2015)			F/M
155	2802	Clonazepam	Yes			0.59	(Takaku et al., 2015)			F/M
156	4913	Procainamide	Yes			1.10	(Takaku et al., 2015)			F/M
157	114976	Norbuprenorphine	Yes			0.49	(Takaku et al., 2015)			F/M
158	446598	Clindamycin	Yes			0.50	(Takaku et al., 2015)			F/M
159	4485	Nifedipine	Yes			0.78	(Takaku et al., 2015)			F/M
160	60815	Remifentanil	Yes			0.72	(Takaku et al., 2015)			F/M
161	1050	Pyridoxal	Yes	0.37	(Giaginis et al., 2009)			C	(Schenker et al., 1992)	C/NC(exp)
162	14052	Ethambutol Hydrochloride	Yes			0.76	(Takaku et al., 2015)			F/M
163	4631	Oxprenolol	Yes			0.37	(Takaku et al., 2015)			F/M
164	13930	Heptachlor Epoxide	Yes			0.34	(Takaku et al., 2015)			F/M
165	60943	SR49059	Yes	0.25	(Hewitt et al., 2007)			C	(Lagrange et al., 2001)	C/NC(exp)
166	2756	Cimetidine	Yes	0.30	(Giaginis et al., 2009)	>0.40	(Howe et al., 1981)			F/M
167	5311010	Atosiban	Yes	0.10	(Valenzuela et al., 1995)			C	(Fullerton et al., 2011)	C/NC(exp)
168	3002977	Maraviroc	Yes	0.26	(Vinot et al., 2013)	0.37	(McCormack and Best, 2014)			F/M
169	4091	Metformin	Yes	0.34	(Giaginis et al., 2009)			C	(Weiner, 2019)	C/NC(exp)
170	44187	Cefoperazone	Yes	0.04	(Giaginis et al., 2009)	0.35	(Takaku et al., 2015)			F/M
171	1051	Pyridoxal 5´-phosphate	Yes	0.07	(Giaginis et al., 2009)			C	(Schenker et al., 1992; Weiner, 2019)	C/NC(exp)

No	CID	Drug (commercial)	Cross	CI[a]	Ref.	F/M	Ref.	C/NC (exp)	Ref.	Criterion
172	135398740	Ganciclovir	Yes	0.17	(Giaginis et al., 2009)			C	(Weiner, 2019)	C/NC(exp)
173	36921	Ticarcillin	Yes	0.04	(Giaginis et al., 2009)	0.91	(Takaku et al., 2015)			F/M
174	62959	Trovafloxacin	Yes	0.19	(Giaginis et al., 2009)			C	(Malek and Mattison, 2010)	C/NC(exp)
175	656511	Mezlocilline	Yes	0.14	(Giaginis et al., 2009)			C	(Weiner, 2019)	C/NC(exp)
176	39031	Sulbenicillin	Yes					C	(Pacifici and Nottoli, 1995)	C/NC(exp)
177	91562	Cefacetrile	Yes			0.50	(Pacifici and Nottoli, 1995)			F/M
178	38103	Cefradine	Yes					C	(Pacifici and Nottoli, 1995)	C/NC(exp)
179	2712	Chlordiazepoxide	Yes					C	(Pacifici and Nottoli, 1995)	C/NC(exp)
180	54680675	Clomocycline	Yes			0.40	(Pacifici and Nottoli, 1995)	C		F/M
181	54707177	Limecycline	Yes					C	(Pacifici and Nottoli, 1995)	C/NC(exp)
182	5959	Chloramphenicol	Yes			0.50	(Pacifici and Nottoli, 1995)			F/M
183	216258	Colistimethate	Yes			0.58[b]	(Pacifici and Nottoli, 1995)			F/M
184	14969	Vancomycin	Yes			0.75	(Pacifici and Nottoli, 1995)			F/M
185	31703	Doxorubicin	Yes					C	(Pacifici and Nottoli, 1995; Weiner, 2019)	C/NC(exp)
186	4914	Procaine	Yes			0.50	(Pacifici and Nottoli, 1995)			F/M
187	2244	Salicylates (Aspirin)	Yes			0.40	(Thiessen et al., 1984)	C	(Jacobson et al., 1991)	F/M
188	135320	N-acetyl-acebutolol	Yes			0.71[b]	(Pacifici and Nottoli, 1995)			F/M
189	3440	Furosemide (Frusemide)	Yes			0.48	(Beermann et al., 1978)			F/M
190	6000	Tubocurarine	Yes					C	(Pacifici and Nottoli, 1995)	C/NC(exp)
191	16078	Tetrahydrocannabinol	Yes					C	(Pacifici and Nottoli, 1995)	C/NC(exp)
192	44814488	11-Nor-Delta-9-Carboxylic Acid-Tetrahydrocannabinol	Yes					C	(Pacifici and Nottoli, 1995)	C/NC(exp)

No	CID	Drug (commercial)	Cross	CI[a]	Ref.	F/M	Ref.	C/NC (exp)	Ref.	Criterion
193	54687	Pravastatin	No			0.09	(Zarek et al., 2013)			F/M
194	65016	Amprenavir	No	0.38	(Giaginis et al., 2009)	0.07	(Gedeon and Koren, 2006)			F/M
195	3035	DDE	No	0.61	(Giaginis et al., 2009)	0.10	(Takaku et al., 2015)			F/M
196	392622	Ritonavir	No	0.09	(Giaginis et al., 2009)	0.12	(Gedeon and Koren, 2006; McCormack and Best, 2014)			F/M
197	441243	Saquinavir	No	0.05	(Giaginis et al., 2009)	~0	(McCormack and Best, 2014)			F/M
198	5362440	Indinavir	No	0.39	(Hewitt et al., 2007)	0.08	(Takaku et al., 2015)			F/M
199	47320	Atracurium Besilate	No			0.10	(Pacifici and Nottoli, 1995)			F/M
200	5314	Suxamethonium	No			0.10	(Pacifici and Nottoli, 1995)			F/M
201	21233	Dimethyl-Tubocurarine (Metocurine)	No			0.08[b]	(Pacifici and Nottoli, 1995)			F/M
202	39765	Vecuronium	No			0.10	(Pacifici and Nottoli, 1995)			F/M
203	439260	Noradrenaline (Norepinephrine)	No			<0.10	(Pacifici and Nottoli, 1995)			F/M
204	16130199	Enfuvirtide	No			~0	(McCormack and Best, 2014)	NC	(McCormack and Best, 2014)	F/M
205	12560	Erythromycin	No			0.03	(Heikkinen et al., 2000)	NC	(Heikkinen et al., 2000)	F/M
206	447043	Azithromycin	No			0.03	(Heikkinen et al., 2000)	NC	(Heikkinen et al., 2000)	F/M
207	5480431	Roxithromycin	No			0.05	(Heikkinen et al., 2000)	NC	(Heikkinen et al., 2000)	F/M
208	3343	Fenoterol	No	0.10	(Giaginis et al., 2009)			NC	(Sodha and Schneider, 1983)	C/NC(exp)
209	3609	Hexoprenaline	No			~0	(Heikkinen et al., 2000)			F/M
210	2083	Salbutamol	No	0.10	(Hewitt et al., 2007)			NC	(Heikkinen et al., 2000)	C/NC(exp)
211	3488	Glibenclamide (Glyburide)	No			0.11	(Mosby, 2017)			F/M
212	11693	Glycopyrrolate	No			0.13	(Murad et al., 1981; Ali-Melkkilä et al., 1990)			F/M
213	441130	Meropenem	No	0.08	(Giaginis et al., 2009)	0.04	(Weiner, 2019)			F/M

No	CID	Drug (commercial)	Cross	CI[a]	Ref.	F/M	Ref.	C/NC (exp)	Ref.	Criterion
214	65028	Oseltamivir (Phosphate)	No	0.13	(Giaginis et al., 2009)			NC	(Weiner, 2019)	C/NC(exp)
215	41693	Sufentanil	No	0.56	(Hewitt et al., 2007)	<0.10	(Sastry, 1999)			F/M
216	84029	Clarithromycin	No			0.08	(Park et al., 2012)			F/M
217	439501	Quabain	No	0.07	(Giaginis et al., 2009)			NC	(Dancis et al., 1983)	C/NC(exp)
218	60835	Duloxetine	No			0.12	(Boyce et al., 2011; Takaku et al., 2015)			F/M
219	118984375	Insulin (MW 5.8 Kda)	No							C/NC(exp)
220	4893	Prazosin	No			0.14[b]	(Pacifici and Nottoli, 1995)			F/M
221	2732	Chlorthalidone	No			0.10[b]	(Pacifici and Nottoli, 1995)			F/M
222	5819	Thyroxine	No			~0.10	(Pacifici and Nottoli, 1995)			F/M
223	5920	Liothyronine	No			~0.10	(Pacifici and Nottoli, 1995)			F/M
224	6024	Cephalothin	Yes			0.40	(Pacifici and Nottoli, 1995; Ward, 1996)			F/M
225	5479529	Cefuroxime	Yes					C	(Craft et al., 1981; Pacifici and Nottoli, 1995)	C/NC(exp)
226	9782	Betamethasone	Yes	0.41	(Giaginis et al., 2009)	0.30	(Pacifici and Nottoli, 1995; Vafaei et al., 2021)			F/M
227	2907	Cyclophosphamide	Yes					C	(Van Calsteren, 2010; Miyamoto et al., 2016)	C/NC(exp)
228	2474	Bupivacaine	Yes	0.73	(Giaginis et al., 2009)	>0.37	(Johnson et al., 1999; Takaku et al., 2015)			F/M
229	175805	Ropivacaine	Yes	0.75	(Giaginis et al., 2009)	0.82	(Johnson et al., 1999)			F/M
230	92727	Lopinavir	No	0.73	(Giaginis et al., 2009)			NC	(Fauchet et al., 2015)	C/NC(exp)
231	213039	Darunavir	No					NC	(Ivanovic et al., 2010; Mandelbrot et al., 2014)	C/NC(exp)

No	CID	Drug (commercial)	Cross	CI[a]	Ref.	F/M	Ref.	C/NC (exp)	Ref.	Criterion
232	2157	Amiodarone	Yes			0.30	(Pacifici and Nottoli, 1995)			F/M
233	90659973	Alcuronium	Yes			0.41	(Ho et al., 1981)			F/M
234	6914273	Dantrolene	Yes			0.40[b]	(Pacifici and Nottoli, 1995)			F/M
235	9433	Aminophylline	Yes			1.08	(Pacifici and Nottoli, 1995)			F/M
236	104774	Desethyl-Amiodarone	Yes			0.40	(Pacifici and Nottoli, 1995)			F/M
237	44814488	11-Nor-Δ^9-tetrahydrocannabinol-9-carboxylic acid	Yes			0.50	(Pacifici and Nottoli, 1995)			F/M
238	60877	Emtricitabine	Yes			>1.20	(McCormack and Best, 2014)			F/M
239	464205	Tenofovir	Yes			0.82	(McCormack and Best, 2014)			F/M
240	6451164	Rilpivirine	Yes			0.74	(McCormack and Best, 2014)			F/M
241	193962	Etravirine	Yes			>0.35	(McCormack and Best, 2014)			F/M
242	54682461	Tipranavir	Yes			0.41	(McCormack and Best, 2014)			F/M
243	54671008	Raltegravir	Yes			1.00	(McCormack and Best, 2014)			F/M
244	5284373	Cyclosporine	Yes					C	(Heikkinen et al., 2000)	C/NC(exp)
245	8982	Nafcillin	Yes					C	(Weiner, 2019)	C/NC(exp)
246	5284603	Oxycodone	Yes					C	(Kokki and Kokki, 2016; Mosby, 2017)	C/NC(exp)
247	2801	Clomipramine	Yes			0.70[b]	(Weiner, 2019)			F/M
248	6291	Mestranol	Yes					C	(Weiner, 2019)	C/NC(exp)

[a] CI Index = Cleareance of drug/ Clearance of antipyrine

[b] Average value

Table S2: Results of five additional runs using the same parameters as in the first solution. The frequency each feature is repeated between partitions is shown in paretheses. Over the five different runs, the following features were repeated, KRFPC566 (5/5), KRFPC608 (4/5) and KRFPC3948 (4/5).

Run	Feature 1	Feature 2	Feature 3	Feature 4	$F_{1/2}$ Train	$F_{1/2}$ Test
1	KRFPC69 (2)	KRFPC566 (2)	KRFPC608 (2)	KRFPC4830 (3)	0.76	0.64
2	KRFPC566 (2)	KRFPC608 (2)	KRFPC3948 (3)	-	0.71	0.61
3	KRFPC566 (3)	KRFPC608 (2)	KRFPC3948 (3)	-	0.71	0.61
4	KRFPC566 (3)	KRFPC608 (2)	KRFPC3948 (2)	-	0.71	0.61
5	KRFPC566 (2)	KRFPC3948 (2)	-	-	0.63	-

Table S3: Results of five additional runs using sets of nine features as individuals in the GA protocol. Over these different genetic algorithms, the following features were repeated, KRFPC3948 (5/5), ROTB (3/5), and KRFPC1653 (2/5).

Run	Feature 1	Feature 2	Feature 3	Feature 4	$F_{1/2}$ Train	$F_{1/2}$ Test
1	KRFPC3948 (2)	KRFPC566 (2)	ROTB (2)	-	0.60	-
2	KRFPC3948 (3)	KRFPC1786 (3)	KRFPC1653 (2)	ROTB(2)	0.56	-
3	KRFPC3948 (4)	KRFPC2714 (2)	KRFPC1653 (2)	-	0.57	0.50
4	KRFPC3948 (3)	KRFPC4531 (2)	-	-	0.48	-
5	KRFPC3948 (3)	KRFPC4755 (2)	ROTB (2)	-	0.50	-

Table S4: Results of five addtional runs using sets of 12 features as individuals in the GA protocol. Over these different GAs, the only repeated feature was KRFPC3948 (3/5).

Run	Feature 1	Feature 2	$F_{1/2}$ Train	$F_{1/2}$ Test
1	KRFPC3848 (2)	ROTB (2)	0.46	-
2	EStateFP75 (2)	-	-	-
3	KRFPC4074 (2)	-	-	-
4	KRFPC3948 (2)	-	-	-
5	KRFPC3948 (2)	-	-	-

Table S5: Results of five additional GA runs changing the cross validation split. Over these different GA runs the following features were repeated: KRFPC566 (4/5), KRFPC435 (2/5), KRFPC3948 (3/5), and KRFPC4830 (3/5).

Run	Feature 1	Feature 2	Feature 3	Feature 4	$F_{1/2}$ Train	$F_{1/2}$ Test
1	KRFPC435 (2)	KRFPC566 (2)	KRFPC3948 (3)	KRFPC4830 (3)	0.81	0.78
2	KRFPC3899 (2)	KRFPC4074 (2)	KRFPC4830 (3)	-	0.64	0.55
3	KRFPC435 (2)	KRFPC566 (3)	KRFPC3948 (2)	-	0.71	0.64
4	KRFPC566 (2)	KRFPC4830 (2)	-	-	0.65	0.54
5	KRFPC566 (2)	KRFPC3948 (2)	-	-	0.63	-

Table S6: Results of five additional GA runs changing the cross validation split with another seed. No features were repeated in the fifth run. Over these different GA runs only the KRFPC608 feature was repeated in three of the five runs.

Run	Feature 1	Feature 2	$F_{1/2}$ Train	$F_{1/2}$ Test
1	KRFPC608 (2)	KRFPC3948 (2)	0.53	0.54
2	KRFPC608 (2)	KRFPC566 (2)	0.53	0.50
3	KRFPC608 (2)		-	-
4	KRFPC3730 (2)	-	-	-
5	-	-	-	-

Table S7: Results of five additional GA runs changing the cross validation split. No features were repeated in the fifth run. Over these different GA runs the following features were repeated: KRFPC435 (3/5) and KRFPC3392 (2/5).

Run	Feature 1	Feature 2	Feature 3	Feature 4	$F_{1/2}$ Train	$F_{1/2}$ Test
1	KRFPC344 (2)	KRFPC435 (2)	KRFPC3737 (2)	KRFPC3392 (2)	0.65	0.63
2	KRFPC435 (2)	KRFPC680 (2)	-	-	0.63	0.64
3	KRFPC608 (3)	KRFPC4830 (2)	-	-	0.63	0.61
4	KRFPC435 (2)	KRFPC3392 (2)	-	-	0.60	0.58
5	-	-	-	-	-	-

Table S8: Results of five additional runs using a different initial population. Over these different genetic algorithms, the following features were repeated: KRFPC566 (3/5), KRFPC3899 (4/5), and KRFPC3948 (4/5).

Run	Feature 1	Feature 2	Feature 3	Feature 4	Feature 5	$F_{1/2}$ Train	$F_{1/2}$ Test
1	KRFPC435 (2)	KRFPC566 (2)	KRFPC3399 (2)	KRFPC3899 (2)	KRFPC3948 (2)	0.80	0.78
2	KRFPC566 (2)	KRFPC3899 (2)	KRFPC3948 (2)	-	-	0.67	-
3	KRFPC3899 (2)	KRFPC3948 (2)	-	-	-	0.47	-
4	KRFPC3899 (2)	KRFPC3948 (2)	-	-	-	0.47	-
5	KRFPC114 (2)	KRFPC566 (2)	-	-	-	0.44	-

Table S9: Results of five additional runs using another initial population. Over these different genetic algorithms, the following features were repeated: KRFPC435 (2/5) and KRFPC566 (2/5).

Run	Feature 1	Feature 2	Feature 3	Feature 4	$F_{1/2}$ Train	$F_{1/2}$ Test
1	KRFPC300 (2)	KRFPC435 (2)	KRFPC3392 (2)	KRFPC3730 (2)	0.60	0.52
2	KRFPC566 (2)	KRFPC4753 (2)	KRFPC4830 (2)	-	0.50	-
3	KRFPC435 (2)	KRFPC3948 (2)	-	-	0.52	0.54
4	KRFPC566 (3)	KRFPC2264 (2)	-	-	0.49	0.46
5	KRFPC413 (2)	KRFPC1638 (2)	-	-	-	-

Table S10: Results of five additional runs using another initial population. No features were repeated in the fifth case. Over these different genetic algorithms, the following features were repeated: KRFPC566 (3/5) and KRFPC669 (2/5).

Run	Feature 1	Feature 2	Feature 3	$F_{1/2}$ Train	$F_{1/2}$ Test
1	dbonds (2)	KRFPC566 (3)	KRFPC669 (2)	0.62	0.61
2	KRFPC435 (2)	KRFPC566 (3)	KRFPC3727 (2)	0.57	0.54
3	KRFPC669 (2)	KRFPC3928 (2)	KRFPC4122 (2)	0.56	-
4	KRFPC566 (3)	KRFPC4830 (3)	-	0.65	0.54
5	-	-	-	-	-

References

Abman, R.P., and Steven (2017). *Fetal and Neonatal Physiology. Fifth Edition.* Elsevier Ltd.

Ali-Melkkilä, T., Kaila, T., Kanto, J., and Iisalo, E. (1990). Pharmacokinetics of glycopyrronium in parturients. *Anaesthesia* 45**,** 634-637.

Allegaert, K., and Van Den Anker, J.N. (2017). "20 - Physicochemical and Structural Properties Regulating Placental Drug Transfer," eds. R.A. Polin, S.H. Abman, D.H. Rowitch, W.E. Benitz, W.W.B.T.F. Fox & P. Neonatal. Elsevier), 208-221.e204.

Ando, M., Saito, H., and Wakisaka, I. (1985). Transfer of polychlorinated biphenyls (PCBs) to newborn infants through the placenta and mothers' milk. *Arch. Environ. Contam. Toxicol.* 14**,** 51-57.

Baker, H., Frank, O., Deangelis, B., Feingold, S., and Kaminetzky, H.A. (1981). Role of placenta in maternal-fetal vitamin transfer in humans. *Am. J. Obstet. Gynecol.* 141**,** 792-796.

Bank, A.M., Stowe, Z.N., Newport, D.J., Ritchie, J.C., and Pennell, P.B. (2017). Placental passage of antiepileptic drugs at delivery and neonatal outcomes. *Epilepsia* 58**,** e82-e86.

Barceloux, D.G. (2012). *Medical Toxicology of Drug Abuse: Synthesized Chemicals and Psychoactive Plants.* Wiley.

Beermann, B., Groschinsky-Grind, M., Fåhraeus, L., and Lindström, B. (1978). Placental transfer of furosemide. *Clin. Pharmacol. Ther.* 24**,** 560-562.

Boyce, P.M., Hackett, L.P., and Ilett, K.F. (2011). Duloxetine transfer across the placenta during pregnancy and into milk during lactation. *Arch. Womens Ment. Health* 14**,** 169-172.

Brown, Walter u., Bell, George c., Lurie, Aron o., Weiss, Jess b., Scanlon, John w., and Alper, Milton h. (1975). Newborn Blood Levels of Lidocaine and Mepivacaine in the First Postnatal Day Following Maternal Epidural Anesthesia. *Anesthesiology* 42**,** 698-707.

Caballero, T., Canabal, J., Rivero-Paparoni, D., and Cabañas, R. (2014). Management of hereditary angioedema in pregnant women: a review. *International journal of women's health* 6**,** 839-848.

Cetin, I., Marconi, A.M., Baggiani, A.M., Buscaglia, M., Pardi, G., Fennessey, P.V., and Battaglia, F.C. (1995). In vivo placental transport of glycine and leucine in human pregnancies. *Pediatr. Res.* 37**,** 571-575.

Cho, N., Fukunaga, K., Kunii, K., and Deguchi, K. (1988). [Bacteriological, pharmacokinetic and clinical studies on the use of ceftriaxone in the perinatal period]. *The Japanese journal of antibiotics* 41**,** 180-195.

Chow, A.W., and Jewesson, P.J. (1985). Pharmacokinetics and safety of antimicrobial agents during pregnancy. *Rev. Infect. Dis.* 7**,** 287-313.

Craft, I., Mullinger, B.M., and Kennedy, M.R. (1981). Placental transfer of cefuroxime. *Br. J. Obstet. Gynaecol.* 88**,** 141-145.

Cressey, T.R., Stek, A., Capparelli, E., Bowonwatanuwong, C., Prommas, S., Sirivatanapa, P., Yuthavisuthi, P., Neungton, C., Huo, Y., Smith, E., Best, B.M., Mirochnick, M., and Team, I.P. (2012). Efavirenz pharmacokinetics during the third trimester of pregnancy and postpartum. *Journal of acquired immune deficiency syndromes (1999)* 59**,** 245-252.

Dancis, J., Kammerman, S., Jansen, V., and Levitz, M. (1983). The effect of ouabain on placental transport of 86Rb. *Placenta* 4**,** 351-359.

Dancis, J., Lehanka, J., and Levitz, M. (1985). Transfer of Riboflavin by the Perfused Human Placenta. *Pediatr. Res.* 19**,** 1143-1146.

Fauchet, F., Treluyer, J.-M., Illamola, S.M., Pressiat, C., Lui, G., Valade, E., Mandelbrot, L., Lechedanec, J., Delmas, S., Blanche, S., Warszawski, J., Urien, S., Tubiana, R., and Hirt, D. (2015). Population Approach To Analyze the Pharmacokinetics of Free and Total Lopinavir in HIV-Infected Pregnant Women and Consequences for Dose Adjustment. *Antimicrob. Agents Chemother.* 59**,** 5727-5735.

Fortunato, S.J., Bawdon, R.E., Swan, K.F., Bryant, E.C., and Sobhi, S. (1992). Transfer of Timentin (ticarcillin and clavulanic acid) across the in vitro perfused human placenta: comparison with other agents. *Am. J. Obstet. Gynecol.* 167**,** 1595-1599.

Freyer, A.M. (2009). Drugs in Pregnancy and Lactation 8th Edition: A Reference Guide to Fetal and Neonatal Risk. *Obstetric Medicine* 2**,** 89-89.

Fujimoto, S., Tanaka, T., and Akahane, M. (1991). Levels of ritodrine hydrochloride in fetal blood and amniotic fluid following long-term continuous administration in late pregnancy. *European Journal of Obstetrics & Gynecology and Reproductive Biology* 38**,** 15-18.

Fullerton, G.M., Black, M., Shetty, A., and Bhattacharya, S. (2011). Atosiban in the Management of Preterm Labour. *Clinical Medicine Insights: Women's Health* 4**,** CMWH.S5125.

Gedeon, C., and Koren, G. (2006). Designing Pregnancy Centered Medications: Drugs Which Do Not Cross the Human Placenta. *Placenta* 27**,** 861-868.

Ghabrial, H., Czuba, M.A., Stead, C.K., Smallwood, R.A., and Morgan, D.J. (1991). Transfer of acipimox across the isolated perfused human placenta. *Placenta* 12**,** 653-661.

Giaginis, C., Zira, A., Theocharis, S., and Tsantili-Kakoulidou, A. (2009). Application of quantitative structure-activity relationships for modeling drug and chemical transport across the human placenta barrier: a multivariate data analysis approach. *J. Appl. Toxicol.* 29**,** 724-733.

Gilstrap, L.C., Bawdon, R.E., Roberts, S.W., and Sobhi, S. (1994). The transfer of the nucleoside analog ganciclovir across the perfused human placenta. *Am. J. Obstet. Gynecol.* 170**,** 967-973.

Grosso, L.M., Triche, E.W., Belanger, K., Benowitz, N.L., Holford, T.R., and Bracken, M.B. (2006). Caffeine Metabolites in Umbilical Cord Blood, Cytochrome P-450 1A2 Activity, and Intrauterine Growth Restriction. *Am. J. Epidemiol.* 163**,** 1035-1041.

Heikkinen, T., Laine, K., Neuvonen, P.J., and Ekblad, U. (2000). The transplacental transfer of the macrolide antibiotics erythromycin, roxithromycin and azithromycin. *BJOG : an international journal of obstetrics and gynaecology* 107**,** 770-775.

Herman, N.L., Li, A.-T., Van Decar, T.K., Johnson, R.F., Bjoraker, R.W., Downing, J.W., and Jones, D. (2000). Transfer of methohexital across the perfused human placenta. *J. Clin. Anesth.* 12**,** 25-30.

Hewitt, M., Madden, J.C., Rowe, P.H., and Cronin, M.T.D. (2007). Structure-based modelling in reproductive toxicology: (Q)SARs for the placental barrier. *SAR QSAR Environ. Res.* 18**,** 57-76.

Ho, P.C., Stephens, I.D., and Triggs, E.J. (1981). Caesarean Section and Placental Transfer of Alcuronium. *Anaesth. Intensive Care* 9**,** 113-118.

Hollier, L.P., Keelan, J.A., Hickey, M., Maybery, M.T., and Whitehouse, A.J.O. (2014). Measurement of Androgen and Estrogen Concentrations in Cord Blood: Accuracy, Biological Interpretation, and Applications to Understanding Human Behavioral Development. *Frontiers in endocrinology* 5**,** 64-64.

Howe, J.P., Mcgowan, W.a.W., Moore, J., Mccaughey, W., and Dundee, J.W. (1981). The placental transfer of cimetidine. *Anaesthesia* 36**,** 371-375.

Ivanovic, J., Bellagamba, R., Nicastri, E., Signore, F., Vallone, C., Tempestilli, M., Tommasi, C., Mazzitelli, L., and Narciso, P. (2010). Use of darunavir/ritonavir once daily in treatment-naive pregnant woman: pharmacokinetics, compartmental exposure, efficacy and safety. *AIDS* 24.

Jacobson, R.L., Brewer, A., Eis, A., Siddiqi, T.A., and Myatt, L. (1991). Transfer of aspirin across the perfused human placental cotyledon. *Am. J. Obstet. Gynecol.* 165**,** 939-944.

Johnson, R.F., Cahana, A., Olenick, M., Herman, N., Paschall, R.L., Minzter, B., Ramasubramanian, R., Gonzalez, H., and Downing, J.W. (1999). A Comparison of the Placental Transfer of Ropivacaine Versus Bupivacaine. *Anesth. Analg.* 89**,** 703.

Kanto, J.H. (1982). Use of Benzodiazepines during Pregnancy, Labour and Lactation, with Particular Reference to Pharmacokinetic Considerations. *Drugs* 23**,** 354-380.

Kokki, H., and Kokki, M. (2016). "Chapter 45 - Central Nervous System Penetration of the Opioid Oxycodone," eds. V.R.B.T.N.O.D.A. Preedy & M. Substance. (San Diego: Academic Press), 457-466.

Kopecky, E.A., Simone, C., Knie, B., and Koren, G. (1999). Transfer of morphine across the human placenta and its interaction with naloxone. *Life Sci.* 65**,** 2359-2371.

Lagrange, F.J., Brun, J.L., Clot, P.F., Leng, J.J., Saux, M.C., Kieffer, G., and Bannwarth, B.G. (2001). Placental Transfer of SR49059 in the Human Dually Perfused Cotyledon In Vitro. *Placenta* 22**,** 870-875.

Lancz, K., Murínová, L., Patayová, H., Drobná, B., Wimmerová, S., Sovčíková, E., Kováč, J., Farkašová, D., Hertz-Picciotto, I., Jusko, T.A., and Trnovec, T. (2015). Ratio of cord to maternal serum PCB concentrations in relation to their congener-specific physicochemical properties. *Int. J. Hyg. Environ. Health* 218**,** 91-98.

Littleford, J. (2004). Effects on the fetus and newborn of maternal analgesia and anesthesia: a review. *Canadian journal of anaesthesia = Journal canadien d'anesthesie* 51**,** 586-609.

Loftus, J.R., Hill, H., and Cohen, S.E. (1995). Placental Transfer and Neonatal Effects of Epidural Sufentanil and Fentanyl Administered with Bupivacaine during Labor. *Anesthesiology* 83**,** 300-308.

Malek, A., and Mattison, D.R. (2010). Drug development for use during pregnancy: impact of the placenta. *Expert Review of Obstetrics & Gynecology* 5**,** 437-454.

Mandelbrot, L., Duro, D., Belissa, E., and Peytavin, G. (2014). Placental Transfer of Darunavir in an Ex Vivo Human Cotyledon Perfusion Model. *Antimicrob. Agents Chemother.* 58**,** 5617-5620.

Mccormack, S.A., and Best, B.M. (2014). Protecting the fetus against HIV infection: a systematic review of placental transfer of antiretrovirals. *Clin. Pharmacokinet.* 53**,** 989-1004.

Miyamoto, S., Yamada, M., Kasai, Y., Miyauchi, A., and Andoh, K. (2016). Anticancer drugs during pregnancy. *Jpn. J. Clin. Oncol.* 46**,** 795-804.

Morselli, P.L., Boutroy, M.J., Bianchetti, G., Zipfel, A., Boutroy, J.L., and Vert, P. (1990). Placental transfer and perinatal pharmacokinetics of betaxolol. *Eur. J. Clin. Pharmacol.* 38**,** 477-483.

Mosby (2017). *Mosby's Drug Reference for Health Professions 6th Edition.* Elsevier Mosby.

Mose, T., Kjaerstad, M.B., Mathiesen, L., Nielsen, J.B., Edelfors, S., and Knudsen, L.E. (2008). Placental Passage of Benzoic acid, Caffeine, and Glyphosate in an Ex Vivo Human Perfusion System. *J. Toxicol. Environ. Health, Part A* 71**,** 984-991.

Murad, S.H.N., Conklin, K.A., Tabsh, K.M.A., Brinkman, C.R.I.I.I., Erkkola, R., and Nuwayhid, B. (1981). Atropine and Glycopyrrolate: Hemodynamic Effects and Placental Transfer in the Pregnant Ewe. *Anesth. Analg.* 60.

Örberg, J. (1977). Placental and Mammary Transfer of Two PCBs (2,4′, 5-TCB and 2,2′, 4,4′, 5,5′-HCB) and Their Effect on Reproductive Capacity in Mice. *Ambio* 6**,** 278-280.

Ormerod, P. (2001). Tuberculosis in pregnancy and the puerperium. *Thorax* 56**,** 494-499.

Pacifici, G.M. (2006). Placental transfer of antibiotics administered to the mother: a review. *Int. J. Clin. Pharm. Ther.* 44**,** 57-63.

Pacifici, G.M., Cuoci, L., Guarneri, M., Fornaro, P., Arcidiacono, G., Cappelli, N., Moggi, G., and Placidi, G.F. (1984). Placental transfer of pinazepam and its metabolite N-desmethyldiazepam in women at term. *Eur. J. Clin. Pharmacol.* 27**,** 307-310.

Pacifici, G.M., and Nottoli, R. (1995). Placental transfer of drugs administered to the mother. *Clin. Pharmacokinet.* 28**,** 235-269.

Paolini, C.L., Marconi, A.M., Ronzoni, S., Di Noio, M., Fennessey, P.V., Pardi, G., and Battaglia, F.C. (2001). Placental transport of leucine, phenylalanine, glycine, and proline in intrauterine growth-restricted pregnancies. *J. Clin. Endocrinol. Metab.* 86**,** 5427-5432.

Park, H.S., Ahn, B.-J., and Jun, J.K. (2012). Placental transfer of clarithromycin in human pregnancies with preterm premature rupture of membranes. *J. Perinat. Med.* 40**,** 641-646.

Pilkington, T., and Brogden, R.N. (1992). Acitretin : A Review of its Pharmacology and Therapeutic Use. *Drugs* 43**,** 597-627.

Rey, E., Giraux, P., D'athis, P., Turquais, J.M., Chavinie, J., and Olive, G. (1979). Pharmacokinetics of the placental transfer and distribution of clorazepate and its metabolite nordiazepam in the feto-placental unit and in the neonate. *Eur. J. Clin. Pharmacol.* 15**,** 181-185.

Roberts, S., Bawdon, R., Sobhi, S., Dax, J., Gilstrap Iii, L., and Wimberly, D. (1995). The maternal-fetal transfer of bisheteroypiperazine (U-87201-E) in the ex vivo human placenta. *Am. J. Obstet. Gynecol.* 172**,** 88-91.

Root, B., Eichner, E., and Sunshine, I. (1961). Blood secobarbital levels and their clinical correlation in mothers and newborn infants. *Am. J. Obstet. Gynecol.* 81**,** 948-956.

Sastry, B.V.R. (1999). Techniques to study human placental transport. *Adv. Drug Delivery Rev.* 38**,** 17-39.

Schenker, S., Johnson, R.F., Mahuren, J.D., Henderson, G.I., and Coburn, S.P. (1992). Human placental vitamin B6 (pyridoxal) transport: normal characteristics and effects of ethanol. *The American journal of physiology* 262**,** R966-974.

Sodha, R.J., and Schneider, H. (1983). Transplacental transfer of beta-adrenergic drugs studied by an in vitro perfusion method of an isolated human placental lobule. *Am. J. Obstet. Gynecol.* 147**,** 303-310.

Sudhakaran, S., Ghabrial, H., Nation, R.L., Kong, D.C.M., Gude, N.M., Angus, P.W., and Rayner, C.R. (2005). Differential bidirectional transfer of indinavir in the isolated perfused human placenta. *Antimicrob. Agents Chemother.* 49**,** 1023-1028.

Takaku, T., Nagahori, H., Sogame, Y., and Takagi, T. (2015). Quantitative structure-activity relationship model for the fetal-maternal blood concentration ratio of chemicals in humans. *Biol. Pharm. Bull.* 38**,** 930-934.

Thakur, A., Harman Kaur, G., Ajay, S., Nipun, M., and Shruti, R. (2011). PHARMACY AND PREGNANCY: A REVIEW. 2**,** 1997-2009.

Thiessen, J.J., Salama, R.B., Coceani, F., and Olley, P.M. (1984). Placental drug transfer in near-term ewes: acetylsalicylic and salicylic acid. *Can. J. Physiol. Pharmacol.* 62**,** 441-445.

Tomson, G., Garle, R.I., Thalme, B., Nisell, H., Nylund, L., and Rane, A. (1982). Maternal kinetics and transplacental passage of pethidine during labour. *Br. J. Clin. Pharmacol.* 13**,** 653-659.

Vafaei, H., Kaveh Baghbahadorani, F., Asadi, N., Kasraeian, M., Faraji, A., Roozmeh, S., Zare, M., and Bazrafshan, K. (2021). The impact of betamethasone on fetal pulmonary, umbilical and middle cerebral artery Doppler velocimetry and its relationship with neonatal respiratory distress syndrome. *BMC Pregnancy and Childbirth* 21**,** 188.

Valenzuela, G.J., Craig, J., Bernhardt, M.D., and Holland, M.L. (1995). Placental passage of the oxytocin antagonist atosiban. *Am. J. Obstet. Gynecol.* 172**,** 1304-1306.

Van Calsteren, K. (2010). Chemotherapy during pregnancy: pharmacokinetics and impact on foetal neurological development. *Facts, views & vision in ObGyn* 2**,** 278-286.

Vinot, C., Gavard, L., Tréluyer, J.M., Manceau, S., Courbon, E., Scherrmann, J.M., Declèves, X., Duro, D., Peytavin, G., Mandelbrot, L., and Giraud, C. (2013). Placental transfer of maraviroc in an ex vivo human cotyledon perfusion model and influence of ABC transporter expression. *Antimicrob. Agents Chemother.* 57**,** 1415-1420.

Ward, R.M. (1996). Pharmacology of the maternal-placental-fetal-unit and fetal therapy. *Progress in Pediatric Cardiology* 5**,** 79-89.

Weiner, C. (2019). *Drugs for Pregnant and Lactating Women 3rd Edition.* Elsevier.

Zarek, J., Degorter, M.K., Lubetsky, A., Kim, R.B., Laskin, C.A., Berger, H., and Koren, G. (2013). The transfer of pravastatin in the dually perfused human placenta. *Placenta* 34**,** 719-721.
